# Exploring the Versatile Uses of Triplet States: Working Principles, Limitations, and Recent Progress in Phosphorescence, TADF, and TTA

**DOI:** 10.1021/acsaom.4c00041

**Published:** 2024-04-19

**Authors:** Larissa G. Franca, David G. Bossanyi, Jenny Clark, Paloma Lays dos Santos

**Affiliations:** † Department of Materials Science and Metallurgy, University of Cambridge, Cambridge CB3 0FS, U.K.; ‡ Department of Physics and Astronomy, University of Sheffield, Sheffield S3 7RU, U.K.; § Department of Electronic and Electrical Engineering, University of Sheffield, Sheffield S1 3JD, U.K.

**Keywords:** Phosphorescence, TADF, TTA, Triplet states, Intersystem Crossing

## Abstract

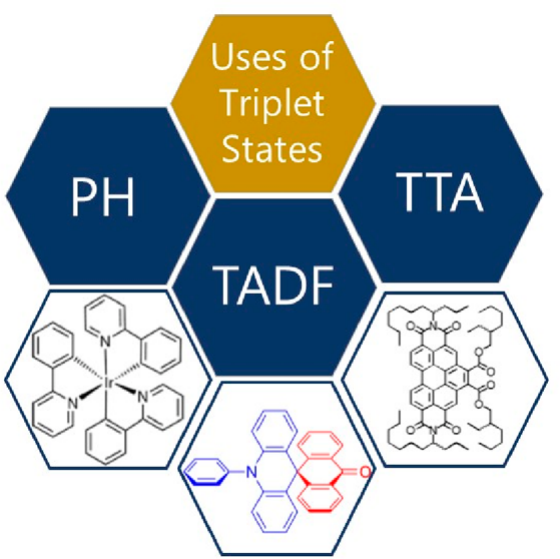

Triplet excited states in organic semiconductors are usually optically dark and long-lived as they have a spin-forbidden transition to the singlet ground state and therefore hinder processes in light-harvesting applications. Also, triplets often cause damage to the system as they can sensitize the formation of reactive singlet oxygen. Despite these unfavorable characteristics, there exist mechanisms through which we can utilize triplet states, and that constitutes the scope of this review. Commencing with an introductory short exploration of the triplet state problem, we proceed to elucidate the principal mechanisms underpinning the utilization of triplet states in organic materials: 1. Phosphorescence (PH), 2. Thermally Activated Delayed Fluorescence (TADF), and 3. Triplet–Triplet Annihilation (TTA). In each section we unveil their working principles, highlight their vast range of applications, and discuss their limitations and perspectives. We dedicate special attention to the use of these mechanisms in organic light-emitting diodes (OLEDs), given that OLEDs represent the most thriving commercial application of organic semiconductors. This review aims to provide readers with insights and opportunities to engage with and contribute to the study of photophysical properties and device physics of organic semiconductors, especially regarding harnessing the potential of triplet states.

## Introduction

Organic semiconductors are active materials in high-end displays,^1,2^ solar cells,^3,4^ biosensors,^5^ lasers,^6,7^ and photosensitizers for therapy.^8^ From biology to lighting technologies, the molecular excited-state behavior is determined by the interplay between photonic, electronic, spin, and nuclear degrees of freedom. Understanding excited-state behavior is key for technology development, in particular, developing strategies to utilize excited states that conventionally exhibit unfavorable characteristics.

Due to the strong electron correlations in most organic semiconductors, the lowest-lying excitonic state is a pure spin-1 triplet exciton state. This state is usually optically dark and long-lived (μs/ms) as it has a spin-forbidden transition to the spin-0 singlet ground state. It also often causes damage to the system as it can sensitize the formation of reactive singlet oxygen (which can react with the molecule, breaking bonds). As such, triplet excitons hinder processes in light-harvesting applications, lasing, and biomedical imaging. In applications that rely on light emission, for example, organic light-emitting diodes (OLEDs), fluorescence microscopy, and lasers, the build-up long-lived triplet population causes degradation and losses. In fluorescence microscopy, despite a low triplet yield (<1%), the problem arises from the triplets generated in fluorescent proteins under continuous illumination.^9^ These triplets produce enough singlet oxygen to harm the delicate biological samples being imaged, thereby significantly restricting the imaging time. Similar problems exist in organic lasers: under continuous wave (CW) illumination triplets prevent lasing,^10^ and achieving lasing under electrical excitation remains challenging due to losses introduced by triplet excitons.^11^ In OLEDs triplets are responsible for the problematic roll-off and degradation at high brightness, and in organic photovoltaics, triplets can be a sub-bandgap loss and degradation pathway.^12−18^

**Figure 1:**
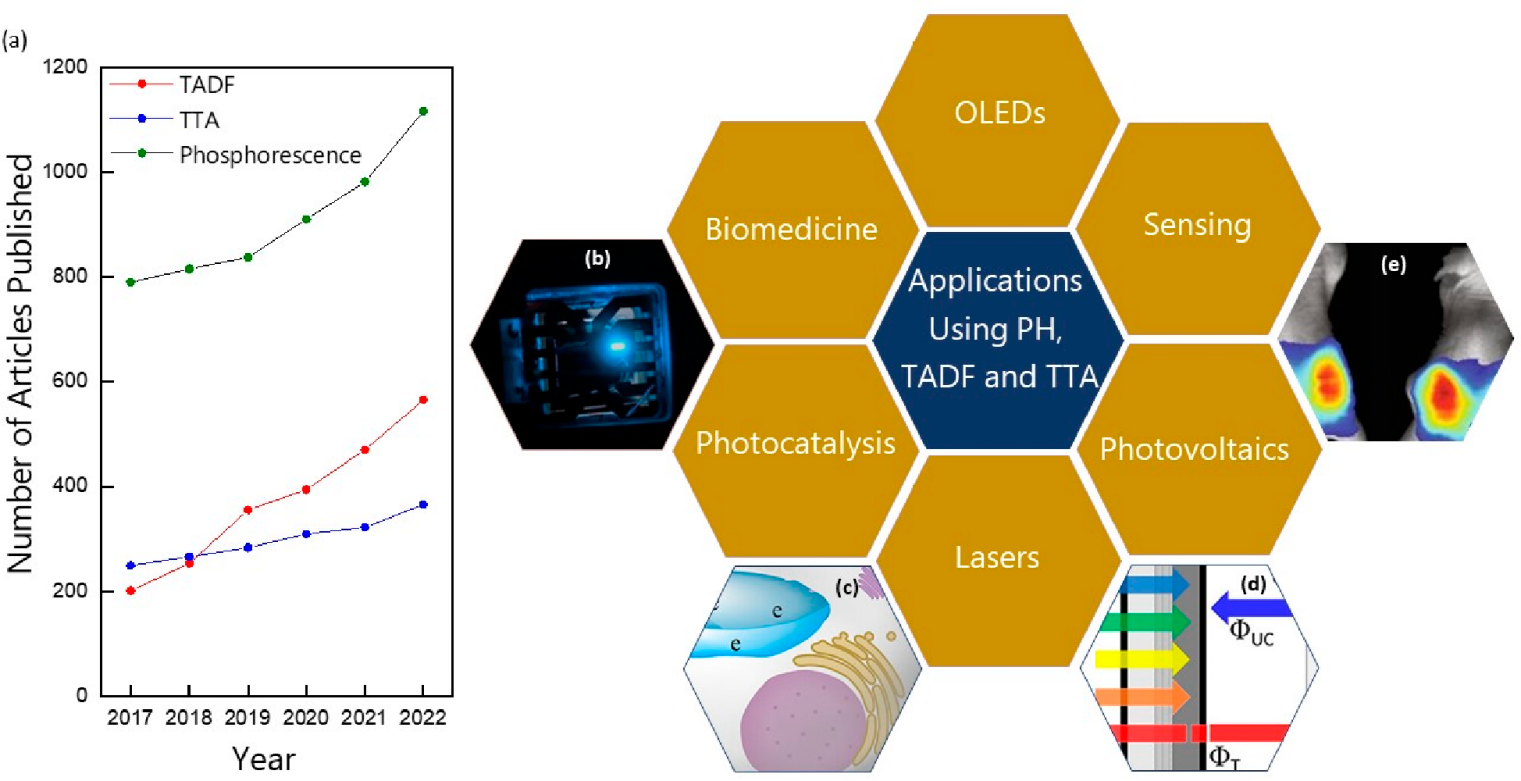
(a) Number of papers published in English per year according to Scopus search for keywords “Phosphorescence”, “TADF”, and “TTA”. Schematic representation of applications using PH, TADF, and TTA. (b) Use of TADF emitters in OLEDs. (c) Use of TADF emitters in photodynamic therapy. Reprinted with permission from ref (19). Copyright © 2023, American Chemical Society. (d) Use of TTA systems in solar cells. Reprinted with permission from ref (20). Copyright © 2021, American Chemical Society. (e) Use of phosphorescence in bioimaging. Reprinted with permission from ref (21). Copyright © 2020, Springer Nature.

To avoid these issues, mechanisms to recycle triplets have been explored in optoelectronic and photonic devices, including phosphorescence (PH), thermally activated delayed fluorescence (TADF), and triplet–triplet annihilation (TTA). While these mechanisms have been known for decades, research in these areas has seen significant growth in the past five years, as indicated by the substantial number of papers published (Figure 1a). Thousands of papers have been devoted to comprehending their fundamental underlying mechanisms, developing new molecular strategies to optimize materials, and exploring their use in different applications.

In this review, we unveil the working principles of PH, TADF, and TTA and explore their versatility in diverse applications including OLED (to which special attention is dedicated due to our expertise in the field), biomedical uses and sensing, as well as other applications like photocatalysis, lasing, transistors, and solar cells. Additionally, we address challenges within each field and propose some potential solutions to open questions. While each mechanism presents unique limitations, there exist overarching challenges common to all three. Below we anticipate and summarize these shared limitations, which are further discussed in different sections.

### Full Understanding of Working Principles

Despite the decades-long exploration and reporting of the working principles of PH, TADF, and TTA, there are still open questions regarding their working principles. Crucial questions, such as the optimal method for calculating reverse intersystem crossing (RISC) rates in TADF systems^22^ or the spin statistical factors in TTA systems,^23^ have not yet been thoroughly explored. Furthermore, the working principles of these systems are influenced by the surrounding molecular environment, complicating the development of universally applicable rules. We highlight that the application of advanced ultrafast optical spectroscopy has proven to be key in conducting detailed studies of these systems, offering valuable insights and answers to many of these unresolved questions.

### Oxygen Elimination

The oxygen molecule, found in both air and water, exists in its triplet state and can quench long-lived states. Typically, ground-state oxygen (triplet state; ^3^O_2_) quenches the excited triplet state by producing singlet oxygen.^24^ This directly impacts the mechanisms targeting harvest triplet states such as PH, TADF, and TTA processes. Consequently, eliminating oxygen is a crucial strategy to enhance the efficiency of these systems and a pivotal step in studying their photophysical properties and advancing their applications. Addressing oxygen elimination is particularly challenging in biomedical applications that involve dispersing these molecules in aqueous solutions, necessitating the development of concepts for oxygen defense and encapsulation.

### Development of Purely Organic Systems

Metal-free materials are cost-effective, abundant, biocompatible, and easily processed. However, they usually show inefficient spin–orbit coupling, hindering intersystem crossing (ISC) and RISC processes key for PH, TADF, and TTA. Works focusing on the development of purely organic molecules that utilize triplet states are certainly increasing; however, PH and TTA systems still strongly rely on scarce metals, which can raise environmental and cost concerns.

### Suitable Solid-State Hosts

Developing appropriate host materials is just as important as the development of PH, TADF, and TTA materials. In the context of OLED applications, designing appropriate hosts, particularly for the blue color, poses a persistent challenge. These hosts must exhibit high triplet energy levels, chemical and thermal stability, and facilitate effective energy transfer to guest molecules. Additionally, hosts with balanced charge carrier mobilities are essential for optimal performance in optoelectronic applications. For TTA, the challenge is enhanced by the need to utilize host systems with high triplet exciton diffusion.

### Device Fabrication Method

Organic-based devices are usually fabricated through a thermal evaporation process, which relies on a high-cost vacuum-dependent method. Moreover, this approach results in a substantial amount of material wastage during evaporation and poses challenges when scaling up production. Conversely, solution-based methods offer a more cost-effective alternative with the potential to produce larger-area devices. Unfortunately, devices produced through solution processing exhibit inferior performance compared to vacuum-deposited devices due to inherent issues related to layer quality and interface layer mixing. Molecular design has proven to be a critical factor in achieving high-performance solution-processed devices, where progress on molecules that show suppressed aggregation formation is very important.

### OLED Efficiency and Lifetime Trade-off

Achieving a balance between device external quantum efficiency (EQE) and operational lifetime is challenging. Strategies that enhance efficiency often involve long-lived molecules that may compromise material stability and lead to a shorter device operational lifetime. Specifically, we emphasize that although the EQE of OLEDs based on TADF and TTA emitters usually achieve theoretical limitations, they still lag behind PH OLEDs in terms of operational lifetime.

We believe that overcoming the key challenges mentioned above with respect to PH, TADF, and TTA systems should set the stage to fully exploit the potential of these fascinating materials. We hope that this review provided readers with insights and opportunities to engage with and contribute to our research area.

## Phosphorescence

The term “phosphorescence” has undergone a few transformations since it became known in ancient times. For an extended period following *Stokes’*(25) introduction of the term “fluorescence” in the mid-19th century, the distinction between fluorescence and phosphorescence relied on the duration of emission after the cessation of excitation. Fluorescence was defined as light emission that ceased simultaneously with the end of excitation, while phosphorescence involved emitted light persisting after excitation ceased. However, this criterion proved inadequate, given the existence of long-lived fluorescence and short-lived phosphorescence as well as other distinct mechanisms of delayed fluorescence. In 1929, *Perrin*(26) stated for the first time that the usual condition for observing phosphorescence is that the excited species passes through an intermediate state before emission. More precisely, nowadays, we say that the phosphorescence phenomenon involves a change in spin multiplicity, characterized by the decay from the triplet excited state to the ground state (T_1_-S_0_). This state can be populated either through intersystem crossing (ISC) from singlet to triplet excited state or via weak direct absorption (S_0_-T_1_), predominantly observed in metal complexes.^27^

### Working Principles

Phosphorescence is a phenomenon of radiative decay of the molecular triplet excited state.^28^ This is the simplest physical process which provides an example of spin-forbidden transition. Below we describe the working principles of phosphorescence in the context of optical excitation, i.e., the processes that follow light absorption. However, in the applications section, we will also discuss phosphorescence that results from electroluminescence and chemiluminescence.

**Figure 2:**
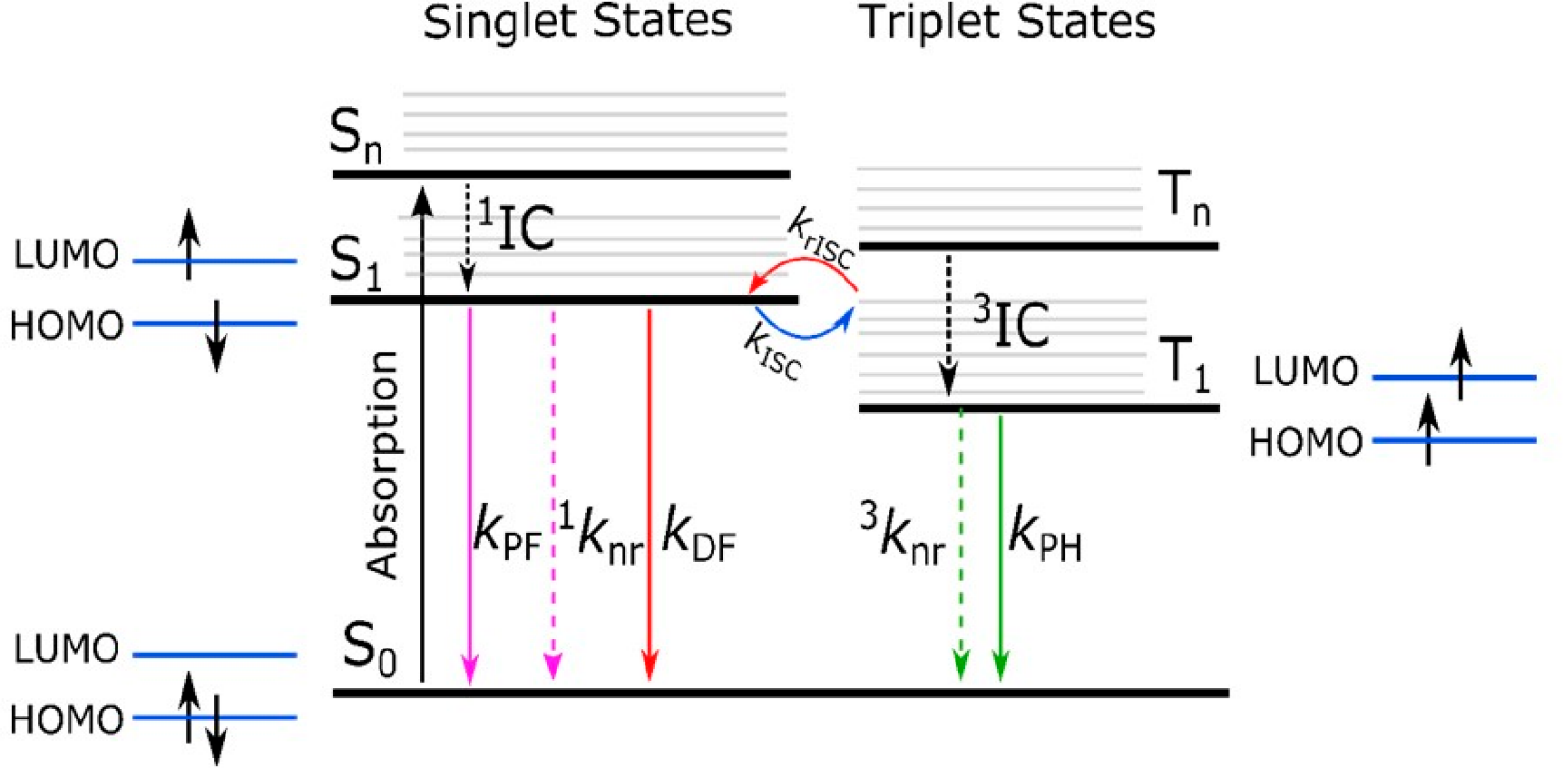
Jablonski diagram. Thicker horizontal lines represent the electronic states, and the thin lines are the vibrational states, where S_0_ is the ground singlet state, S_1_ is the first excited singlet state, and S_n_ are higher excited singlet states, T_1_ is the first excited triplet state, and T_n_ are higher excited triplet states. The spin configurations of S_0_, S_1_, and T_1_ are also shown in the diagram. Note this is a simplified diagram.

Figure 2 shows a typical Jabłoński diagram, illustrating the electronic states and processes in organic molecules. This diagram provides a simplified representation of electronic energy levels, without accounting for energy dispersion as a function of spatial variation, such as nuclear distances. The thicker horizontal lines represent the electronic states, and the thin lines are the vibrational states, where S_0_ is the ground singlet state, S_1_ is the first singlet excited state, S_n_ is the higher singlet excited state, T_1_ is the first triplet excited state, and T_n_ is higher triplet excited states. The spin configurations of the ground state, S_1_, and T_1_ are also shown.

Upon light absorption, electrons are promoted to S_n_. Usually, they relax to S_1_ rapidly (10^–12^ s or less) without photon emission (nonradiative process), a transition called internal conversion (^1^IC). This process is then followed by three distinct processes: i. radiative emission yielding prompt fluorescence (PF), a fast decay component (pico-nanosecond range) with rate constant assigned as *k*_PF_; ii. nonradiative internal conversion (IC), ^1^*k*_nr_; or iii. intersystem crossing (ISC) to the triplet states.

While PF and IC occur between states of the same multiplicity, ISC is a nonradiative, adiabatic process that occurs between states of different multiplicity. The spin rule says that optical transitions with change in the spin multiplicity are forbidden (Δ*S* = 0), but ISC can become allowed by coupling between the particles’ spin and their orbital angular momenta (spin–orbit coupling, SOC). Since a large SOC allows a large ISC rate, SOC in organic molecules will be effective in inducing transitions between spin states if one (or both) of the electrons involved approaches a “heavy” atom (as the process is stronger for larger nuclei, SOC ∝ Z^4^). This is because the heavy atom nucleus is capable of causing the electron to accelerate, thereby creating a strong magnetic moment as the result of increased orbital motion. Thus, the strength of the SOC coupling varies depending on the atomic number of the atoms, and therefore heavy atoms (e.g., Br, Pb, Pt) induce a strong SOC leading to fast ISC rates. Even for light atoms, the topology of the involved orbitals also plays a significant role, which can also lead to SOC (as seen in purely organic systems, for example). An illustration of this phenomenon is the transition from nπ to ππ* observed in benzophenone.

Regardless of the magnitude of SOC, to induce a transition between states of different spin, the total angular momentum of the system (orbit plus spin) must be conserved. Thus, a transition from a singlet spin to a triplet spin is compensated by a transition from a p orbital of an orbital momentum 1 to a p orbital of angular momentum 0, i.e., p_*x*_ → p_*y*_ transition.

Apart from SOC, hyperfine coupling (HFC) can also contribute to ISC between singlet and triplet states. This mechanism arises from interactions between an electron’s spin and nucleus’ spin in the same molecule or in bimolecular systems. Some experimental studies have proposed that hyperfine coupling induces ISC, and the interplay of SOC and HFC contributions was evaluated.^29−31^ For a more advanced theoretical chemical perspective on ISC we recommend papers by *Marian et al*.^32−34^ Once the triplet states are populated, they can either recombine to the ground state by radiative emission, phosphorescence (*k*_PH_), or by nonradiative processes (^3^*k*_nr_), or by a further spin flip, back to the singlet excited state, by reverse intersystem crossing (*k*_RISC_). Although phosphorescence is formally forbidden, it can be activated in molecules with sufficient SOC. However, due to this formally forbidden nature of the transition, the rate constants for triplet emission are several orders of magnitude smaller than those for fluorescence. For a more in-depth theory of the PH phenomenon, we guide the readers to the review paper from *Agren et al*.,^35^ which highlights the most important achievements in the theory and computations of phosphorescence and related spin-forbidden phenomena.

Regarding investigating the photophysical properties of phosphorescent materials, it is important to highlight that PH is highly influenced by factors such as molecular aggregation, temperature, and exposure to air. Particular care needs to be taken to avoid oxygen in the samples, e.g., by degassing solutions or measuring films under vacuum or under nitrogen flow, when determining phosphorescence spectra, efficiencies, and lifetimes. Typically, high-intensity phosphorescence spectra are collected at low temperatures, which can be achieved using cryogenic conditions, for example, by using liquid nitrogen. Unfortunately, these conditions pose challenges to the practical application of phosphorescent materials and limit their feasibility for technological uses.

### Applications

#### OLEDs

One of the most successful applications of phosphorescent materials is as emissive materials in OLED displays.^36^ Over the past decade, OLEDs have spearheaded a revolution in displays, establishing themselves as the preferred choice for mobile phone screens and high-end TVs.^37^ Commercial OLED displays use phosphorescent emitters to produce green and red light. The selection of phosphorescent emitters is strategic, driven by the fact that 75% of the excitons generated in OLEDs are triplets and 25% singlets. Therefore, it becomes essential to employ materials that show efficient emission from triplet states. This choice results in devices exhibiting internal quantum efficiency (IQE) of up to 100%. However, a significant challenge persists in the generation of blue light. The absence of a stable blue phosphorescent material remains a major challenge, and usually blue PH OLEDs show extremely short operational lifetime. Consequently, commercial OLEDs resort to fluorescent emitters for generating blue light, resulting in considerable energy inefficiency in displays.^38^ Trying to tackle this issue, many works dedicated to optimizing blue PH OLED have been reported,^39−42^ and, recently, *Kim et al*.^43^ reported the longest device lifetime reported for blue PH OLED. Their device showed CIE (Commission Internationale de l’éclairage) *y* < 0.20 and an excellent lifetime of LT_70_ = 1113 h (the time to reach 70% at the initial luminance of 1000 cd m^–2^). They achieved this performance by introducing bulky 3,5-di-*tert*-butyl-phenyl into the N-heterocyclic carbene moiety in the Pt(II) complex, enhancing the photochemical stability of the high-lying metal-centered triplet state and preventing undesirable host–guest interactions, factors that contributed to a longer device lifetime and higher color purity.

Concerning molecular design, the most efficient materials demonstrating robust phosphorescence typically involve complexes featuring a heavy metal center, such as Ru (ruthenium), Rh (rhodium), Os (osmium), Ir (iridium), Pt (platinum), and Au (gold). These materials exhibit rapid ISC facilitated by the pronounced SOC effect. The first example of a phosphorescent emitter was the metal complex PtOEP, reported by *Thompson and Forrest*(44) in 1998. This emitter was used as a dopant for red OLEDs, and an external quantum efficiency (EQE) of 4% was achieved. Since this pioneering work, an impressive amount of research effort has been devoted to materials optimization based on platinum(II).^45^ The second important breakthrough by *Thompson and Forrest*(46) was in 1999, when they reported Ir(ppy)3, the most studied green Ir(III) complex. Over the years, Ir(III) complexes have stood out as promising candidates for OLEDs as they offer easy chemical modifications, tunable photophysical properties, and strong stability.^47,48^ We highlight works by *Ma and Wong*(49) and *Zheng and Huang*(50) where they explored novel emitters systematically in order to achieve material optimization and color control, and we guide the readers to the review by *Pan et al*. for exploration of more Ir(III) complexes.^51^

Regarding PhOLED device engineering, devices having ultrathin light-emitting layers (<1 nm) have been widely explored in monochromatic and white OLEDs.^52−54^ These ultrathin layers (usually undoped) offer advantages such as a simplified device structure and preparation process, greater flexibility in design, reduced material consumption, and optimal utilization of excitons.

#### Biomedical Use and Sensing

Exploring the photophysical properties of phosphorescent organometallic compounds induced by photoexcitation has opened numerous applications, particularly in biomedical uses. These compounds offer tunable excitation and emission wavelengths across the entire visible (even near-infrared) spectrum, significant Stokes shifts (often >5000 cm^–1^), and long lifetimes.^55^ This makes them of great interest as a class of materials for bioimaging, guided disease treatment and drug delivery, and sensors for detecting oxygen, pH, and other analytes. *Williams et al*.^56^ reported one of the first examples of time-resolved imaging based on phosphorescent platinum(II) complexes as luminescent probes with emission with a maximum of around 520 nm. However, the transparency window of biological tissues occurs approximately between 700 and 950 nm.^57^ Consequently, the development of phosphors capable of extending the optical range to cover this window has become highly appealing. This poses a significant challenge due to the energy gap law, wherein nonradiative transitions increase as the emission gap decreases.^58^

**Figure 3:**
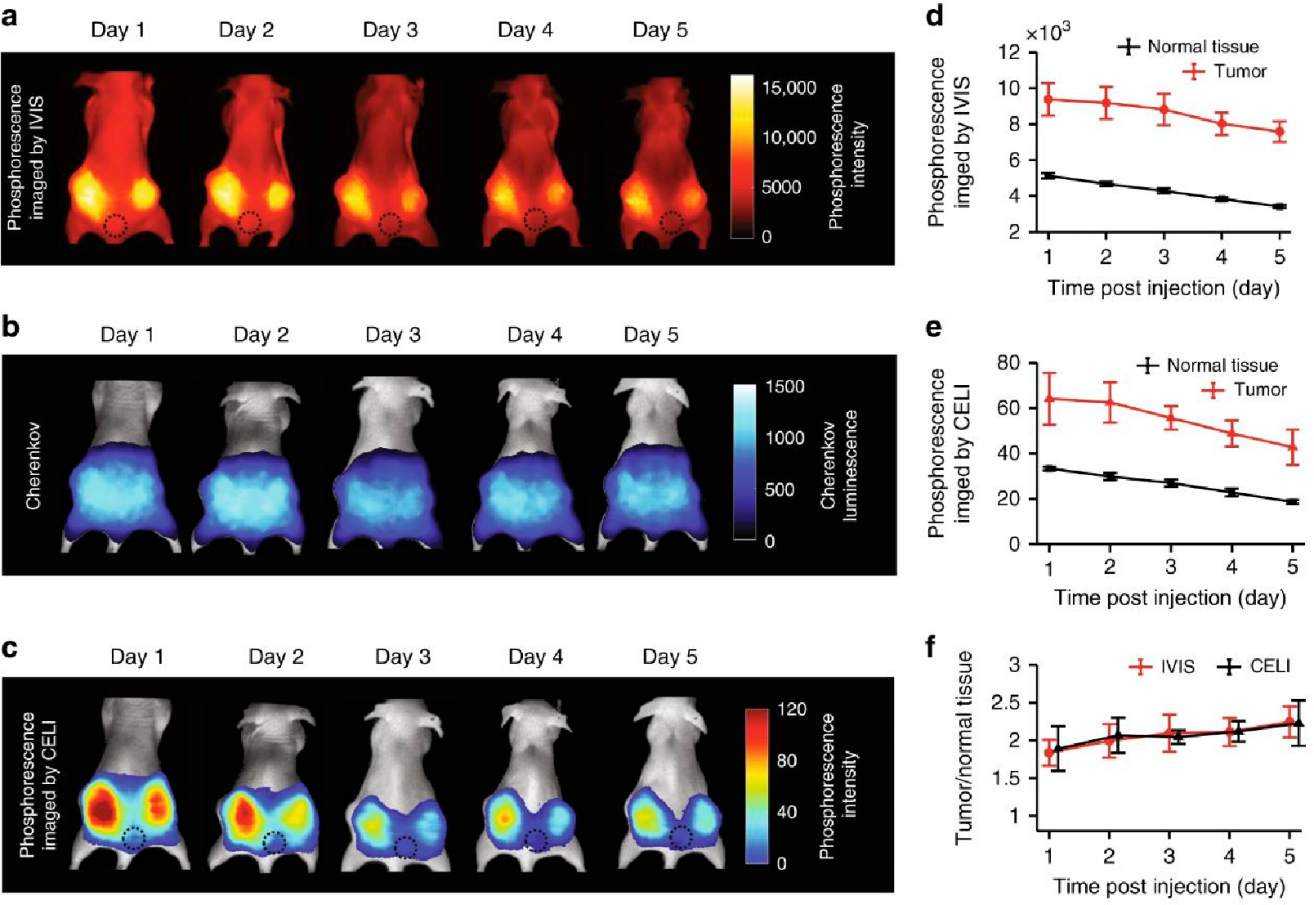
Use of near-infrared (NIR) phosphorescence in bioimaging. (a) Imaging of phosphorescence intensity. (b) Imaging of Cherenkov-excited phosphorescence intensity. (c) Phosphorescence intensity images obtained through CELI over a 5-day period following injection of Pt(II) complexes, along with corresponding histograms. Average intensity of the phosphorescence acquired by IVIS (d) and CELI (e) for the tumor area and for the normal tissue (*n* = 4). (f) The ratio of the signals tumor/normal tissue as imaged by IVIS and CELI (*n* = 4). Error bars represent standard error of the mean. Reprinted with permission from ref (21). Copyright © 2020, Springer Nature.

Substantial progress has been achieved for Pt(II) and Ir(III) complexes emitting into the NIR region.^59−61^ Notably, *Czerwieniec et al*.^62^ proposed a strategy involving the variation of Pt complex nuclearity and the position within the ligand as a highly efficient tool for modulating and fine-tuning the emission properties. Crucially, these accomplishments have been extended to include in vivo mapping. For instance, *Pogue et al*.^21^ investigated the utilization of a phosphorescent Pt complex to monitor dynamic oxygen levels in tumors throughout the course of treatment (shown in Figure 3). The authors also discussed the impact of signal intensity over time, and we encourage the readers to refer to the paper for a more detailed explanation of this topic.

Another crucial role of organometallic complexes is their function as photosensitizers, effectively involving the absorption of light and initiating energy transfer or redox processes.^63^ The triplet excited state of these organomettalic photosensitizers can directly transfer its energy to molecular oxygen, generating singlet oxygen or causing photochemical reactions.^64^ This is particularly important in photodynamic therapy, which involves the activation of a photosensitizer with a light source to produce reactive species that can kill neighboring target cells. As PH photosensitizers display high ISC rates upon optical excitation the high density of formed triplet excitons can interact with molecular oxygen (^3^O_2_), producing its excited-state singlet form ^1^O_2_ but also producing other reactive oxygen species. These reactive oxygen species are cytotoxic and thus attack nearby cells.^65^ An example of this application is demonstrated by *Wong et al*.,^66^ who developed a new photosensitizer agent by conjugating a cyclometalated Ir(III) complex with a xanthene dye to create a mitochondria-targeting photosensitizer involved in singlet oxygen formation. This compound exhibited an excellent photodynamic therapy effect in killing tumors in mice. Additional examples and advancements in these biomedical applications can be found in the referenced reviews.^67−70^

Beyond these applications, it is worth mentioning that organometallic compounds have also found application in the field of chemiluminescence. A recent example, as published by *Qi et al*.,^71^ involved the utilization of an Ir(III) solvent complex to develop an electrogenerated chemiluminescence sensor capable of distinguishing bases in oligonucleotides relevant in clinical diagnoses.

#### Other Applications

An alternative to PhOLED devices involves the use of phosphorescent metal complexes in the design of light-emitting electrochemical cells (LECs). These devices typically have much simpler structures and need less severe encapsulation compared to OLEDs.^72^ The mechanism in LECs involves the transport of mobile ions between electrodes in response to applied electrical stimuli.^73^ The pioneering example of an LEC was published by *Wightman et al*.^74^ in 1996, and significant progress has been made since then, with *Carmichael et al*.^75^ publishing a fully stretchable LEC in 2012.

As these materials function as photosensitizers, they hold significant potential in energy upconversion (UC) for solar cell technologies and photocatalysis. Illustrating examples in the photocatalysis field, *Schlau-Cohen et al*.^76^ demonstrated a biohybrid catalyst comprising the photosynthetic light-harvesting protein and multiple conjugated [Ru(bpy)_3_]^2+^ photocatalysts. The photocatalyst demonstrated significant improvements beyond increased yields and reactivity. It is environmentally sustainable, exhibiting activity under low-energy irradiation, and is easily reusable. In photovoltaics, phosphorescent materials find application as both the interface layer and the active layer as well as dopants to enhance transport performance.^77^ A recent illustration of this is demonstrated by *Wang et al*.,^78^ who utilized an organometallic complex as a cathode interlayer to reduce the metal work-function. By density functional theory calculations and surface characterizations, the authors show that the organometallic complexes that contain anions and cations are prone to form anion–cation dipoles on the metal surface, hence drastically reducing the metal’s work function. This innovation led to a significant enhancement in the power conversion efficiency of inverted perovskite solar cells, achieving an impressive value of 21%.

Additionally, due to their exhibited versatility and high responsiveness to stimuli of organometallic phosphors, manifesting variations in their photophysical properties and also displaying electrochemical redox ability, LECs have potential applications in data encryption, data security protection, and rechargeable batteries.^79−81^

#### Limitations & Perspectives

One fundamental limitation in the field is to achieve room-temperature phosphorescence in metal-free materials, due to inefficient SOC and an easily-quenched radiation relaxation process. Nonetheless, there is a preference for metal-free materials as they are generally cost-effective, biocompatible, and easily processed. Two main principles to achieve efficient room-temperature phosphorescence in purely organic materials are (i) enhancing ISC efficiency by using aromatic carbonyl, heavy-atom, or/and heterocycle/heteroatom-containing compounds;^82−85^ (ii) suppressing intramolecular motion and intermolecular collision which can quench excited triplet states, e.g., embedding phosphors into polymers and packing them tightly in crystals.^86−89^*Gallardo et al*.^90^ recently proposed an alternative approach for the latter, demonstrating that by partially shielding the localization of the emissive triplet state at the molecular core—surrounded by three bulky donor units—it likely enhances the strong SOC between them, enabling room-temperature phosphorescence to manifest even in a solution.

Similarly, progress in metal-free phosphorescent materials also holds promise for expanding their applications in biomedicine. Despite metal complexes displaying relatively low toxicity, there is a lack of reported studies on their long-term biosafety. This necessitates a careful exploration of potential cytotoxicity in aromatic π-systems and cyclo-coordinated metal cores, along with their long-term physiological effects, to advance clinical applications.

In the most explored cases of organometallic complexes, metals such as iridium or platinum are present, and these are rare and very expensive. Hence, there is a need to delve into more abundant metals and explore their photophysical properties, as these properties depend heavily on the chosen metals. Take phthalocyanine complexes, for instance; challenges can arise from the synthesis, demanding either high temperatures or expensive catalysts.

## Thermally Activated Delayed Fluorescence (TADF)

Historically, the discovery of delayed fluorescence marked a significant milestone in the field of optical spectroscopy. It was initially observed in the eosin molecule by *Delorme and Perrin*(91) in 1929 and further characterized by *Lewis and Kasha*.^92^ This molecule inspired the name of the mechanism as E-type delayed fluorescence, although it is now known as the “thermally activated delayed fluorescence” mechanism. In 1961, *Parker and Hatchard*(93) proposed the mechanism as it is understood today. Subsequently, numerous studies have focused on identifying and investigating this effect in various molecules. Nonetheless, a report by *Adachi et al*.,^94^ published in 2011, had a profound impact on the use of this class of molecules, highlighting their potential for significantly enhancing OLED efficiency.

### Working Principle

Generally speaking, the TADF mechanism facilitates the upconversion of triplet excited states into singlet excited states through RISC, owing to the very small energy gap between them (typically below 0.2 eV) and sufficient thermal energy to promote equilibrium between the singlet and triplet excited states.^95−97^ Consequently, the TADF mechanism is highly dependent on temperature, and due to its longer lifetime (driven by the triplet excited state), both TADF and phosphorescence are very susceptible to quenching, such as by molecular oxygen.^98,99^ The RISC rates that drive the TADF mechanism can be described as
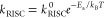
1where *k*_RISC_^0^ is a pre-exponential factor, *E*_a_ is the activation energy for the TADF process, *k*_B_ is the Boltzmann constant, and *T* is temperature. Through this equation, an important plot known as the Arrhenius plot can be obtained, allowing the estimation of the activation energy of the process. For most of the TADF cases, the *E*_a_ can be directly correlated to the Δ*E*_ST_.^100^

The energy of the singlet and triplet excited states can be described in terms of the exchange energy (eq 2,3). Consequently, the energy splitting between the singlet and triplet excited state (Δ*E*_ST_) can be estimated as twice the exchange energy (eq 4), calculated by eq 5.
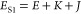
2
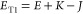
3
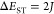
4
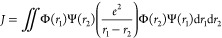
5Here, Φ and Ψ represent the HOMO and LUMO wave functions, *r*_1_ – *r*_2_ is the spatial separation of the HOMO and LUMO, and *e* is the electron charge. To minimize the Δ*E*_ST_ it is necessary to minimize the exchange energy. Two main parameters can lead to *J* ≈ 0: (i) reducing the overlap between Φ and Ψ wave functions, i.e., HOMO and LUMO in the molecule, and (ii) increasing the separation distance between the two electrons.

A typical molecular design that can meet these requirements and result in a TADF molecule involves incorporating a donor group (electron-rich, D) attached to an acceptor group (electron-deficient, A). As shown by *Dias et al*.,^101^ linking these units in a nearly orthogonal relative orientation achieves spatial separation between the HOMO and LUMO and, consequently, minimizes Δ*E*_ST_. Because of this orthogonal D–A relative orientation, these units are decoupled and behave independently from each other, forming their own excited states, namely, locally excited (LE) states. Additionally, by interacting weakly with each other, these units also form charge transfer (CT) states.

**Figure 4:**
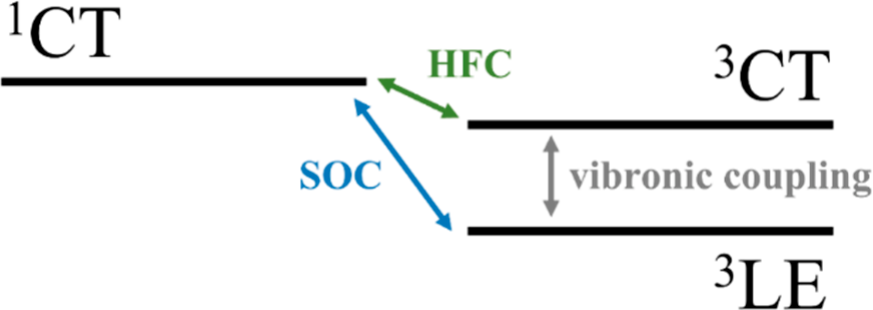
Representation of the coupling interactions between the electronic states involved in the TADF mechanism.

In the case of these D–A and/or D-A-D types of TADF molecules, which possess strong CT excited states, the singlet and triplet excited states involved in the mechanism are the ^1^CT and ^3^CT, respectively. However, as *Lim et al*.^102^ demonstrated, SOC coupling between singlet and triplet states with the same spatial orbital occupation is formally zero. Hence, a more complex second-order perturbation theory is required to describe the TADF mechanism, which considers not only spin–orbit coupling but also vibronic coupling and hyperfine coupling.^103^Figure 4 represents the coupling interactions between the electronic states involved in the TADF mechanism. To enable the spin-flip and facilitate the TADF mechanism in these D–A and/or D-A-D types of TADF molecules, *Monkman et al*.^104^ proposed that a third mediating state, such as locally excited triplet state (^3^LE), is required. The energy of this intermediary triplet state should be close to that of ^3^CT/^1^CT in order to allow for efficient vibronic coupling with the ^3^CT and for spin–orbit coupling with ^1^CT.

Therefore, the impact of the ^3^LE states led to a more general expression for the RISC rates, described by *Penfold et al*.^105^ (eq 6).
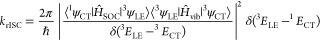
6Here, the number π is divided by the Planck’s constant (ℏ). The first term considers the coupling between the ^1^CT and ^3^LE states via SOC. Additionally, the SOC term incorporates the hyperfine coupling, involving the interaction between the ^3^CT and the ^1^CT, using the ^3^LE as a mediator state. The second term in the equation considers vibronic coupling, which drives the interaction between the ^3^LE and ^3^CT states. The vibronic coupling plays a crucial role in the ISC/RISC mechanism, by effectively mixing the ^3^LE and ^3^CT states. This mixing, as initially described by *Lim et al*.,^102^ is significant because, as mentioned before, it facilitates the transition to ^1^CT by SOC, considering that the transition from ^1^CT to ^3^CT is degenerate and consequently has zero SOC.

Regarding molecular design, various D and A groups have been employed over the years to synthesize these D–A and/or D-A-D types of TADF molecules and evaluate their potential.^106−111^ In reviews by *Dias et al*.^112^ and *Huang et al*.,^113^ nitrogen-based donor units, e.g., carbazole, diphenyl amine, phenoxazine, acridine, and their derivatives, are shown to be the most commonly employed donor units. Within the category of acceptor units, a wide range has been explored, including nitrogen heterocycles, benzophenones, cyanobenzenes, diphenylsulfones, and their derivatives.^114^

**Figure 5:**
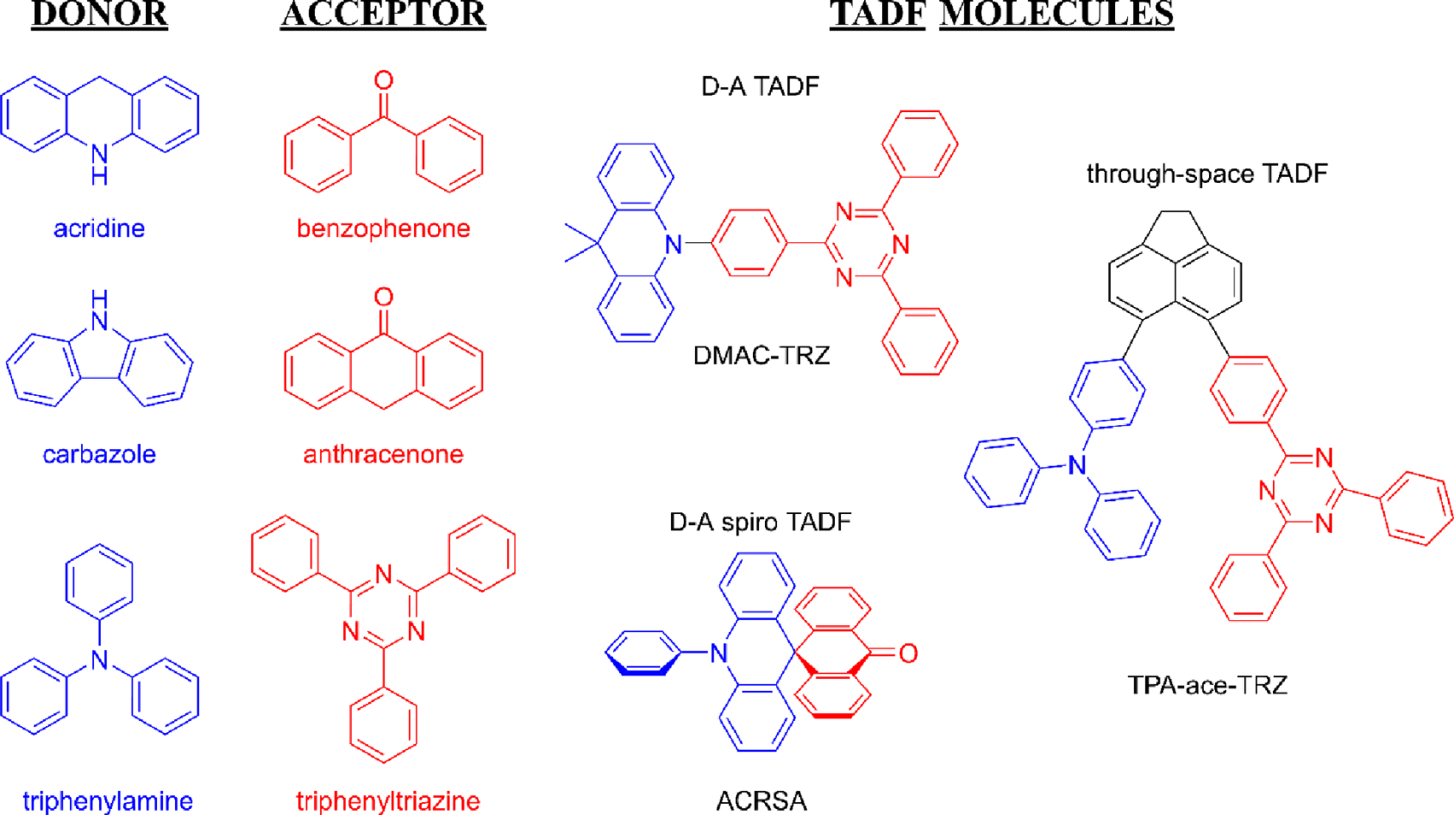
Molecular structure of the typical donor (blue) and acceptor (red) groups and different structures of TADF molecules. Donor units: acridine, carbazole, and triphenylamine (TPA). Acceptor units: benzophenone, anthracenone, and triphenyltriazine (TRZ). TADF molecules: DMAC-TRZ (9,9-dimethyl-9,10-dihydroacridine), ACRSA (10-phenyl-10*H*,10′*H*-spiro[acridine-9,9′-anthracen]-10′-one), and TPA-ace-TRZ (4,4′-(1,2-dihydroacenaphthylene-5,6-diyl)bis(*N*,*N*-diphenylaniline).

Due to the importance of the relative orientation of the D–A units in the Δ*E*_ST_ and, consequently, RISC rates, the choice of the linkage also plays a crucial role in the design of the TADF molecules. There are several ways to connect those units, for example: by C–N bond, by the spiro bridging, or even using neutral spacers and bridges. The majority of TADF molecules reported are C–N linked, allowing relative rotation between the D and A. To control and restrict torsional motion between the D and A in these C–N linked TADF materials, various strategies were proposed. For example, *Bryce et al*.^115^ suggested attaching heavy adamantyl groups, and *Lee et al*.^116^ propose the linkage of a diphenyltriazine acceptor in carbazole donors. Spiro TADF molecules, on the other hand, have a rigid and orthogonal bridge, which enforces the restriction of D–A motion through the tetrahedral configuration of the sp^3^ hybridized spiro carbon atom.^117^ The pioneering example, spirobifluorene D–A molecules, was published in 2012 by *Adachi and co-workers*,^118^ inspiring subsequent examples detailed in *Zysman-Colman et al*.^119^ This rigid and orthogonal spirocarbon bridging bond between the donor and acceptor units critically decouples them, leading to highly complex photophysical properties. We have explored these properties in the case of the ACRSA molecule (Figure 5) both in solution and solid-state films.^120−122^ Another alternative linkage involves the use of an inert scaffold/bridge which increases D–A separation and gives rise to the so-called through-space TADF molecules.^123^ In these materials, the D and A units are positioned further apart within the molecules, and this molecular structure leads to the formation of intramolecular through-space CT states (TSCT). *Kaji et al*.^124^ reported that this innovative design can achieve high RISC rates. It is worth mentioning that the TADF mechanism in the TSCT molecules can be understood in a similar manner to that of TADF exciplexes, wherein intermolecular CT states are formed by physically blending D and A molecules.^125−128^Figure 5 shows examples of TADF molecular structures representative of each of these types mentioned above. The TADF molecules (DMAC-TRZ, ACRSA, TPA-ace-TRZ) were initially reported by *Chih Wu et al*.,^129^*Adachi et al*.,^130^ and *Zysman-Colman et al*.,^131^ respectively.

Apart from these typical TADF molecules, researchers have also extended these structures to design of TADF polymers and macromolecules (e.g., dendrimers),^114,132−134^ aiming to improve the capability of solution-processing materials, potentially reduce processing cost, and enhance suitability for large-scale applications.^135−137^ Among the examples, *Hudson et al*.^133^ designed a TADF dendimer based on the BPPZ acceptor substituted with dendritic donors, while *Yang et al*.^138^ first proposed a side-chain engineering strategy for the development of TADF polymers in 2016. Another important and broad category of TADF molecules includes organometallic complexes,^139^ mostly driven by the strong SOC. Predominant examples revolve around Cu (I) complexes,^140−143^ among which *Yersin and coauthors*(144) have presented an efficient TADF mechanism in a dinuclear Cu(I) complex. There is a growing number of reports involving other metals, such as Ag(I),^145,146^ Au(I),^147,148^ Pd(II),^149,150^ and, more recently, Pt(II).^151^

Lastly, there is an emerging class called multiple resonance (MR) TADF molecules. *Hatakeyama’s group*(152) has been at the forefront of designing this type of molecules, exemplified by the synthesis of one of the most significant molecules in this category, v-DABNA.^152^ MR TADF molecules are designed to achieve HOMO and LUMO separation in plane by strategically placing donor and acceptor atoms in different regions of the molecule within one plane. This arrangement creates an alternating electron density distribution pattern over the molecular structure, i.e., the HOMO electron density is 90° out of phase with that of the LUMO.^153,154^ For these molecules, the TADF mechanism also operates via a second-order spin-vibronic coupling mechanism,^155^ which occurs through upper-triplet crossings from thermally populated T_n_ states back to the emissive S_1_.^156^

**Figure 6:**
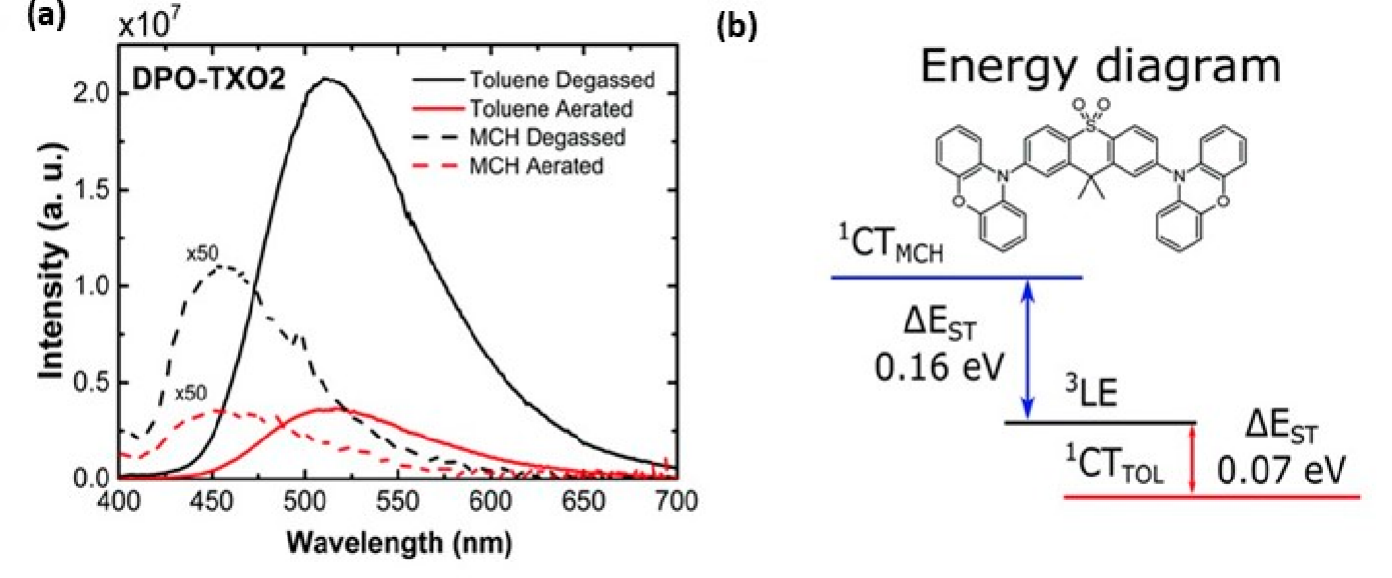
Environment control of energy splitting between singlets and triplets. (a) Photoluminescence (PL) spectra of DPO-TXO2 in MCH and toluene in degassed and aerated solutions. (b) Chemical structure of DPO-TXO2 and the energy-level arrangement for both solutions. Adapted and reprinted with permission from ref (157). Copyright © 2016, The Royal Society of Chemistry.

Similarly to the importance of molecular design in TADF molecules, the impact of the environment on their properties is significant. The presence of charge transfer (CT) states in the majority of TADF molecules makes them highly sensitive to their local environment. We^157^ have demonstrated that the environment can be used to control the Δ*E*_ST_ by studying the molecule DPO-TXO2, a D–A–D TADF emitter formed by phenoxazine donors and the 9,9-dimethylthioxanthene-*S*,*S*-dioxide (TXO_2_) acceptor. This is expected because CT and LE states exhibit different responses to changes in the environment due to their very different polar characters as shown in our work.^157^Figure 6 shows the ^1^CT emission spectra of DPO-TXO2 in methylcyclohexane (MCH) and toluene solutions. The energy difference between these two emission spectra gives rise to two distinct scenarios; in MCH the ^1^CT is located above the ^3^LE state, and in toluene the ^1^CT is located below the ^3^LE. The ^3^LE emission in DPO-TXO2 comes from the donor unit. Thus, the magnitude of the Δ*E*_ST_ energy values for MCH and toluene were identified to be 0.16 and 0.07 eV, respectively. As a direct consequence of the difference in Δ*E*_ST_ value, the DF emission contribution to the overall emission was different in each solvent. This analysis was made by comparing the emission intensity in aerated and degassed solutions. The ^1^CT emission increases by a factor of 3.10 (MCH) and 4.8 (toluene) when oxygen is removed. Thus, the contribution of DF is 52% and 82% for MCH and toluene, respectively. However, it is important to notice that the most efficient case would be a third case, where all states involved in the RISC process are degenerate (^1^CT, ^3^CT, and ^3^LE) with Δ*E*_ST_ equal to zero. Moreover, studies by *Zheng et al*.^158^ show that nonpolar solvents can promote the TADF process in a triple hydrogen-bonded triquinolonobenzene molecule, while polar solvents suppress it. This aligns with observations by *Adachi et al*.^159^ for carbazole benzonitrile derivatives, emphasizing that the addition of polar solvents is not always beneficial.

In solid films, environmental effects become more complex. TADF emitters in neat films may experience quenching in their emission properties due to aggregate formation, leading to nonradiative losses.^160−162^ Consequently, selecting appropriate host molecules for TADF is crucial to avoid quenching and control electrical transport properties.^163^ The host molecules’ static dielectric constant, polarizability, rigidity, and packing properties play a significant role. Experimental observations^164^ and exploration by a parametrized *ab initio* model by *Painelli et al*.^165^ highlight that the host environment can directly affect dihedral angles between donor and acceptor units, immobilizing the molecules in various conformations, strongly impacting Δ*E*_ST_. The conformational effects and, hence, the distribution Δ*E*_ST_ result in time-dependent spectral shifts and multiexponential components in emission decay. Analyzing these phenomena can be challenging; a methodology based on a Laplace transform fitting of delayed fluorescence to unveil these distributions directly and extract them as a density of rates has been recently proposed.^166^ Various types of host molecules, including polymers and small molecules, have been used for TADF molecules. When designing the host, several criteria must be fulfilled, such as having a high triplet energy level, chemical and thermal stability, and balanced charge carrier mobilities.^167^

### Applications

#### OLEDs

As mentioned before, *Adachi et al*.^94^ reported the first OLED using a TADF emitter in 2011. Since then, significant attention has been directed toward enhancing their efficiency, establishing OLEDs as one of the most crucial applications of TADF molecules. Here, we present a few examples within this category, while more detailed information can be found in the references.^167−169^

Remarkable progress has been made in terms of efficiency in recent years.^170−174^*Zhao et al*.^175^ recently reported highly efficient blue and deep-blue OLEDs, with external quantum efficiencies of 43% and 41%, and Commission Internationale de l’Eclairage coordinates (CIEx, y) of (0.14, 0.18) and (0.14, 0.15), respectively, making them among the most efficient blue OLED devices.

On the field of white OLEDs, *Zhao et al*.^176^ employed multifunctional TADF materials for both hosts and emitters, enhancing power efficiency and achieving an outstanding white OLED with an efficiency of 31%. TADF molecules have also been investigated for circularly polarized OLED, and in 2021, *Chen et al*.^177^ proposed the use of TADF as a sensitizer to fabricate high-efficiency circularly polarized OLED with an efficiency achieving 21% at 1000 cd m^2^.

Moreover, TADF-OLEDs suffer from severe device degradation (especially in the blue color) because relatively long-lived triplet excitons in TADF molecules directly affect the operational stability and efficiency roll-off characteristics of TADF-OLEDs. These effects are observed because of the increase of exciton deactivation processes at high current density, including exciton–exciton and exciton-polaron interactions.

Concerning operational stability, numerous studies have highlighted the important role of molecular design in achieving a long operational lifetime for OLEDs. *Tang et al*.^178^ recently demonstrated that deuteration of the acceptor, in addition to a deuterated donor, can boost the device lifetime from 15 h (deuterated donor only) to 23 h (deuterated donor and acceptor). Regarding roll-off, *Adachi et al*.^179^ presented a deep-blue TADF emitter that, owing to its extremely fast exciton lifetime of 750 ns, minimizes efficiency roll off in TADF-OLEDs, once more highlighting the role of careful molecular design on device performance.

While most of the devices presented above are vacuum-processed OLEDs, significant progress has been made in solution-processed OLEDs, offering advantages for the fabrication of low-cost, large-area, and flexible displays for commercial production. In this regard, TADF polymers^180−183^ are very attractive materials as they are compatible for spin coating, inkjet printing, and roll-to-roll coatings fabrication methods. TADF polymers are classified according to the position of TADF active units in the polymer structure, and the five main types are (1) Core-acceptor/Shell-donor dendritic TADF polymer; (2) Main-chain TADF polymer; (3) Backbone-donor/Pendant-acceptor TADF polymer; (4) Side-chain TADF polymer; (5) Self-emission TADF polymer. *Wang at al*.^184^ summarized these different TADF polymers design strategies and discussed the latest works utilizing them. They also pointed out the challenges of TADF polymer-based devices, highlighting the certain unavoidable defects in the device layers, which makes the polymers more difficult to optimize, with unreproducible results compared to small molecules-based devices.

Regarding device performance using TADF polymers, we highlighted work by *Yambem et al*.,^185^ which introduced a TADF polymer with carbazole and α-carboline pendants as the emissive layer in a solution-processed flexible inkjet-printed TADF OLED. With a very simple structure, they achieved an inkjet-printed OLED on a glass substrate that resulted in high luminance of ≈9000 cd m^–2^. Moreover, recently, *Wang et al*.^186^ achieved a significant milestone in the stretchability of TADF OLEDs. They synthesized a polymer that achieves a stretchability of 125%, with an external quantum efficiency of 10%, demonstrating a fully stretchable OLED.

#### Biomedical Uses and Sensing

In the field of biomedicine, purely organic TADF molecules exhibit significant promise due to their photophysical properties, extended lifetimes, and excellent biocompatibility arising from their metal-free molecular structure.^187,188^ Notable contributions include pioneering work by *Peng et al*.^189^ in 2014, who introduced a TADF molecule derived from fluorescein for time-resolved fluorescence microscopy imaging of cancer cells. In 2020, *Hu et al*.^190^ reported a TADF emitter with bacterial 16S rRNA-targeting ability for diagnosing bacterial infections. *Hudson and co-workers* integrated TADF-active materials into water-soluble polymer dots in 2021,^191^ facilitating near-infrared immunofluorescent labeling of human breast cancer cells. Most recently, *Fu et al*.^192^ demonstrated high-performance TADF nanoparticles for super-resolution imaging in living cells in 2023.

**Figure 7:**
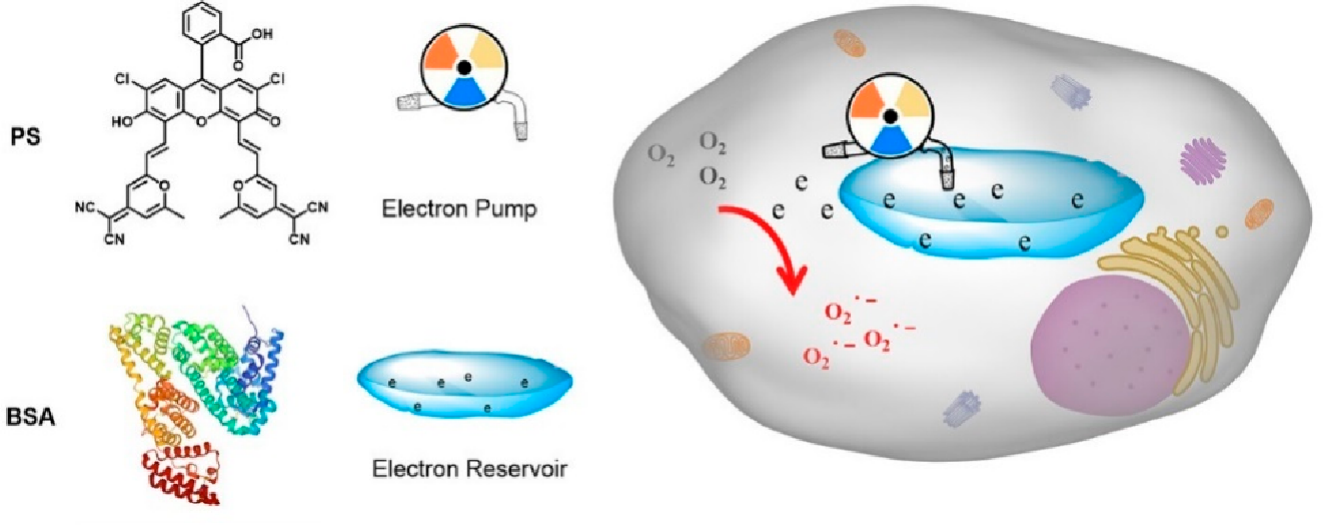
Use of TADF materials in photodynamic therapy. Schematic diagram of the TADF photosensitizer PS acting as an “electron pump” and BSA as an “electron reservoir”. In essence, PS, functioning as a TADF photosensitizer, act as an electron pump due to its enhanced electron transfer capacity. This photosensitizer acts to pump electrons from the BSA electron reservoir and continuously supplies a constant flow of electrons to O_2_, generating the superoxide anion radicals (O_2_·^–^), a highly toxic reactive oxygen species crucial for photodynamic therapy. The PS@BSA system was shown to have excellent tumor cell killing in *in vivo* experiments. Reprinted with permission from ref (19). Copyright © 2023, American Chemical Society.

TADF molecules have been also explored in the context of photodynamic therapy, and we highlight the work by *Lee and colleagues*,^193^ which introduced water-dispersible TADF nanoparticles, presenting the first metal-free organic photosensitizer for oxygen formation. Later, *Peng et al*.^19^ enhanced the efficiency of photodynamic therapy for hypoxic tumors by utilizing a TADF photosensitizer as an “electron pump”. They used bovine serum albumin (BSA) as an “electron reservoir” to encapsulate the TADF photosensitizer PS, and the integrated roles of the PS@BSA system showed excellent tumor-killing effect for tumor-bearing mice in the in vivo experiments (Figure 7). Moreover, *Zhang et al*.^194^ developed metal-free NIR TADF nanophotosensitizers with improved tissue penetration depth for photodynamic therapy, demonstrating their performance at both the cell and small animal levels.

Given that the TADF mechanism relies on triplet excited states with long lifetimes and is highly dependent on temperature, oxygen and temperature sensing has attracted significant interest. The first report using the TADF mechanism for an oxygen sensor was proposed by *Wolfbeis et al*.^195^ in 2013, using ^13^C_70_ fullerene in polymer hosts. *Hudson and co-workers*(196) extended this in 2020, publishing red-emissive polymers exhibiting TADF as thermoresponsive materials for sensors. Researchers have further explored biomodal sensing, where the TADF mechanism can be used as a dual-responsive method, sensing simultaneous oxygen and temperature, as demonstrated by *Zhang et al*.^197^ and *Borisov et al*.^198^ More in-depth information on this topic can be found in referenced reviews.^187,199−201^

#### Other Applications

TADF molecules are also utilized as photocatalysts due to their lower toxicity profiles and widespread availability compared to mostly used metal complexes. Various types of photoinitiators exhibiting efficient TADF are under investigation, including metal complexes and purely organic molecules, as demonstrated by studies conducted by *Lalevée et al*.^202^ and *Milsmann et al*.^203^ According to *Zysman-Colman et al*.,^204^ 2,4,5,6-tetra(carbazol-9-yl)benzene-1,3-dicarbonitrile, known as 4CzIPN and first reported by *Adachi et al*.^205^ in 2012, is among the most widely studied purely organic TADF-based photocatalysts.

Moreover, TADF has recently drawn attention to pumped organic solid-state lasers.^206^ Promising results have been demonstrated in studies by *Liao et al*.^207^ and *Zhao et al*.^208^ using TADF microcrystals for this purpose. In the realm of transistors, *Namdas et al*.^209^ achieved high-efficiency light-emitting field-effect transistors based on a solution-processed TADF emitter. Finally, we also highlight the use of TADF materials in the field of solar cells, where *Chou et al*.^210^ introduced this year a new and practical approach that exploits thermally activated delayed fluorescence molecules. These molecules serve as photosensitizers, storage units, and signal transducers, ultimately optimizing solar thermal energy storage.

#### Limitations & Perspectives

**Figure 8:**
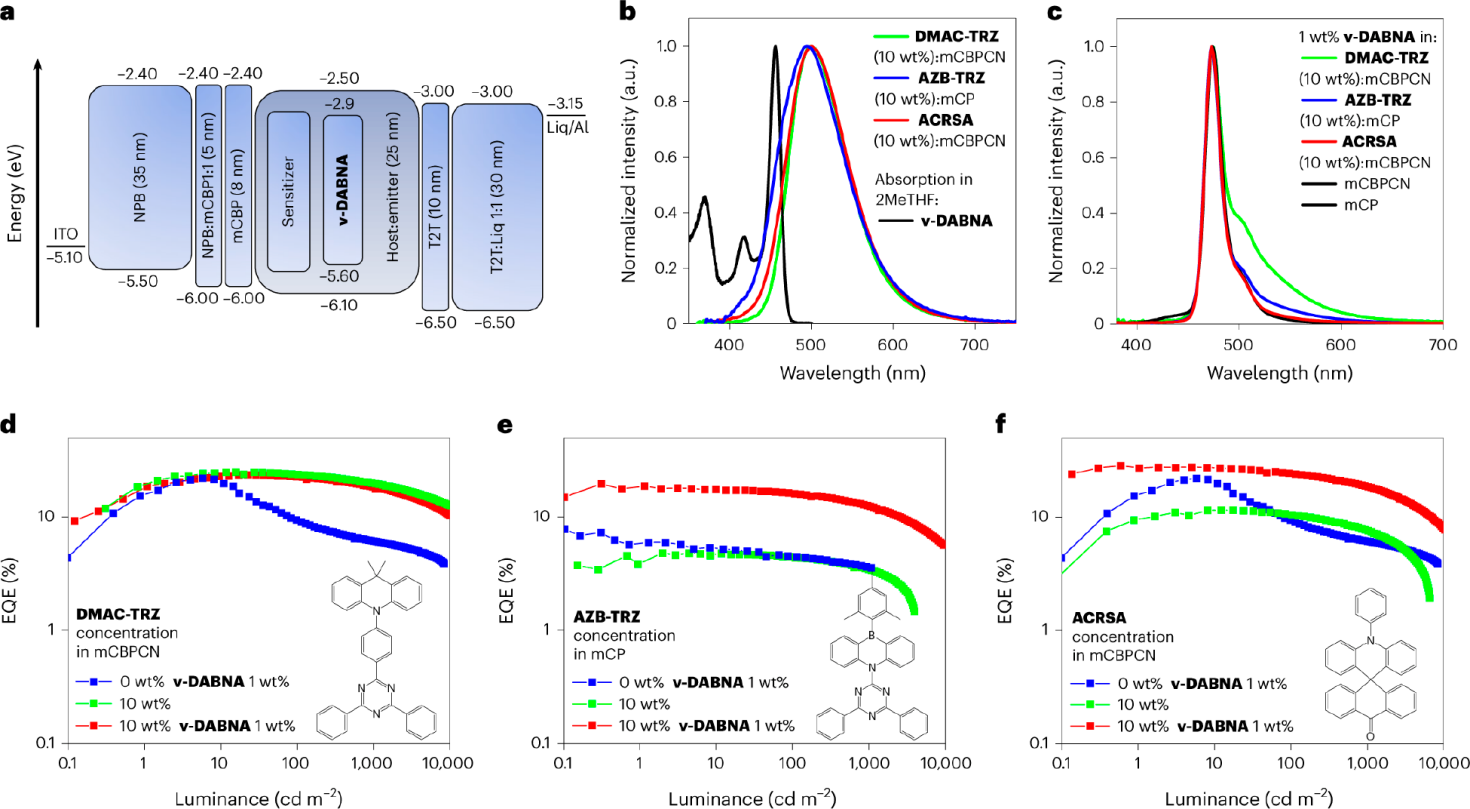
HF OLEDs. (a) Device architecture and (b) EL spectra of TADF devices and (c) HF-OLEDs. A virtually complete FRET is observed in the ACRSA HF-OLEDs that have the EL spectra identical to those of ν-DABNA. Conversely, residual sensitizer emission is observed (on the red side of the EL emission) in the DMAC-TRZ and AZB-TRZ HF-OLEDs. (d) EQE vs luminance of DMAC-TRZ, (e) AZB-TRZ, and (f) ACRSA OLEDs with and without the terminal emitter (blue curve for ν-DABNA without the presence of the sensitizer is shown for comparison). Reprinted with permission from ref (214). Copyright © 2024, Springer Nature.

When discussing OLED technology, TADF molecules face several challenges, particularly in improving color purity, addressing efficiency roll-off and low-stability issues, and ensuring the availability of suitable host materials, especially in blue TADF OLEDs. In response to these challenges, in particularly targeting achieving high EQE and high color purity, hyperfluorescence (HF) OLEDs, introduced by *Adachi et al*. in 2015,^211^ offer a promising solution. This approach employs a mix of three materials in the emissive layer, facilitating Förster resonance energy transfer (FRET) from the singlet excited state of the TADF material to the fluorescent emitter, resulting in high color purity emission^211−213^ (narrowband electroluminescence). In this field, we highlight *Kwon et al*.^213^ HF systems, utilizing TADF host materials like pMDBA-DI and mMDBA-DI, along with a pure blue MR-TADF *t*-Bu-ν-DABNA. This work achieved impressive results with enhanced device efficiency, narrow emission, and long operational lifetimes; however, it is still not sufficient to meet industrial standards. Importantly, most examples of HF have limited their scope to only the “best” performing TADF materials as sensitizers. However, recently *Monkman et al*.^214^ showed that HF can transform intrinsically “poor” TADF emitters into blue HF-OLEDs with exceptional performance. This work demonstrated that blue HF-OLEDs utilizing a greenish sensitizer exhibit a remarkable tripling of external quantum efficiency (∼30%) compared with non-HF devices (Figure 8). It is worth noting the challenges in HF fabrication, attributed to the intricate process of simultaneously evaporating three materials and dealing with very low doping ratios, making it a particularly demanding task.

Another strategy to enhance device roll-off and stability involves achieving fast emission rates (suppressing long-lived species involved in degradation mechanisms) by designing molecules that show RISC between higher triplet states and singlet states (T_n_ → S_m_; *n* ≥ 2, *m* ≥ 1). Despite Kasha’s rule suggesting that high-lying excited states have minimal effect on fluorescence, hot exciton materials typically exhibit a large T_n_–T_1_ energy gap and a small T_n_–S_m_ energy splitting. The former inhibits IC from T_n_ to T_1_, while the latter can increase the RISC rate from T_n_ to S_m_.^215,216^

In recent years, many hot exciton materials have been utilized in OLEDs, primarily in blue, deep-red, and NIR colors, with limited reports on green materials. While these materials often surpass the spin statistical efficiency limit, their EQE generally remains below 10%. The controversial nature of the hot exciton approach arises from the reliance on theoretical calculations for determining energy levels in upper-level triplet states, lacking effective photophysical experiments to substantiate the mechanism. Questions persist regarding the role of molecular packing orientation on substrates or TTA in achieving the theoretical EQE limit, but recent reports are beginning to provide experimental evidence^217^ for the mechanism, such as works by *Wang*.^218^

Another significant challenge in the TADF field is the design of suitable hosts (especially for blue color), requiring high triplet energy levels, chemical and thermal stability, and balanced charge carrier mobilities.^219^ Strategies proposed by researchers, such as *Strohriegl et al*.^220^ and *Blinco et al*.,^221^ aim to develop efficient and stable hosts for blue-emitting OLEDs. Host molecules also have an impact on TADF molecule stability; *You et al*.^222^ attributed this to conformeric heterogeneity in solid films, potentially favoring spontaneous bond dissociation in certain conformers as opposed to others.

The development of TADF materials for biomedicine and sensing is still in the early stages, presenting a range of challenges and opportunities. Inherent properties such as poor water solubility, targeting ability, and large-scale production require careful consideration. A significant challenge involves achieving a prolonged luminescence lifetime, which can be accomplished by suppressing quenching mechanisms.^187^ TADF-containing polymer nanoparticles offer a promising solution to these challenges, as they are highly effective, water-dispersible, and resistant to quenching. However, challenges persist in the size-control procedures during the preparation of these nanoparticles.^199^

Finally, we highlight the scarcity of TADF materials emitting in the deep-red to near-infrared region, essential for optimal transparency in biological tissue. This obstacle may be overcome by employing planar MR TADF materials, which are scarce with only few works reported.^223,224^

## Triplet–Triplet Annihilation

During the 1960s, many reports presented observations of delayed fluorescence within solutions containing aromatic hydrocarbons, specifically anthracene, phenanthrene, and pyrene.^225,226^ The mechanism was understood as an excited dimer, with its intensity being directly proportional to the square of the rate of absorption of exciting light.^227^ Subsequently, with the elucidation of the mechanism involving the influence of thermal activation (the TADF mechanism), it was proposed to refer to the mechanism of delayed fluorescence resulting from triplet–triplet quenching as pyrene-type or P-type delayed fluorescence, nowadays known as triplet–triplet annihilation (TTA).^228^ Remarkably, despite the mechanism having been elucidated as early as the 1960s, it was not until 1998 that the utilization of TTA to enhance the efficiency of fluorescent OLEDs was proposed.^229^ Several years later, a limited number of studies reported the observation of TTA in electroluminescence devices,^230,231^ leading to a substantial increase in attention toward the development of these organic materials for OLEDs and solar cells.^232,233^

### Working Principle

Triplet–triplet annihilation (TTA) is a bimolecular process in which the collision of two molecules in triplet excited states can lead to their annihilation, providing sufficient energy to enable one of them to return to the S_1_ state. *Merrifield and co-workers* developed a kinetic scheme to describe triplet–triplet annihilation, which they formulated as^234^
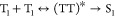
7

The intermediate, denoted as (TT)*, creates a triplet-pair state, wherein the triplets are in close enough proximity to interact. The nature of these triplet-pair states has been, and continues to be, a subject of considerable uncertainty and debate.^235−239^ Due to its bimolecular nature, TTA is typically observed in concentrated solutions and exhibits a strong dependence on the diffusivity of the triplet excitons.^240,241^

**Figure 9:**
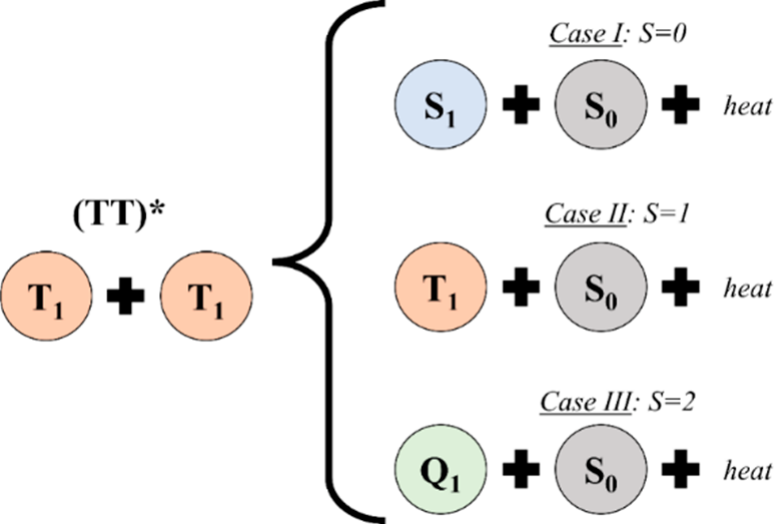
Schematic representation summarizing the three possible cases resulting from the combination of two triplet excited states.

Combining two triplet excited states can not only give rise to singlet states. The total spin angular moment (*S*) must be conserved, resulting in three different cases depending on their spin (Figure 9). When the spin of both triplet excited states is opposite, the resulting state is a singlet state (*S* = 0). If the energy provided approaches or exceeds that of the S_1_ state, the electron undergoes relaxation to the lowest singlet excited states (S_1_ state). Similar to the description of the PH and TADF mechanism, when electrons are in the S_1_ state, they may undergo radiative/nonradiative decays from the S_1_ state (^1^*k*_r_^F^/^1^*k*_nr_) and ISC populating the triplet excited state (T_1_). By decaying radiatively, it gives rise to delayed fluorescence, as the singlet excited state will emit with a longer lifetime. If the total spin is 1 (*S* = 1), it means that the combination leads to the nonradiative decay of one of the triplets. In theory, there is a potential for the formation of a quintet state (*S* = 2) by spin-conservation during the combination of two triplet excited states; however, it has not yet been observed. Therefore, the collision likely leads to scattering of the two triplet excited states. In the cases of molecular crystals, it has been shown that the absence of quintet states is attributed to their higher energies compared to the cumulative energy of two triplet excited states.^242^

An important distinguishing feature between delayed fluorescence originating from TADF or TTA is that, in the TTA mechanism, the DF intensity exhibits a characteristic quadratic dependence on excitation light intensity, owing to its nature as a bimolecular recombination. Taking this into consideration, the DF intensity at low excitation densities is given by eq 8
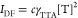
8where *c* represents the fraction of triplet–triplet pairs that generate a singlet state, and *γ*_TTA_ is the rate constant for the radiative singlet decay. [T] represents the concentration of triplet excitons. Additionally, [T] can be also described by the conventional rate equation under steady state, as shown in eq 9.^243^
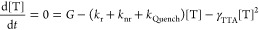
9Here, *G* denotes the formation rate of triplet excitons, while *k*_r_ and *k*_nr_ represent the radiative and nonradiative decay rates, respectively, and *k*_Quench_ is the monomolecular quenching rate.

**Figure 10:**
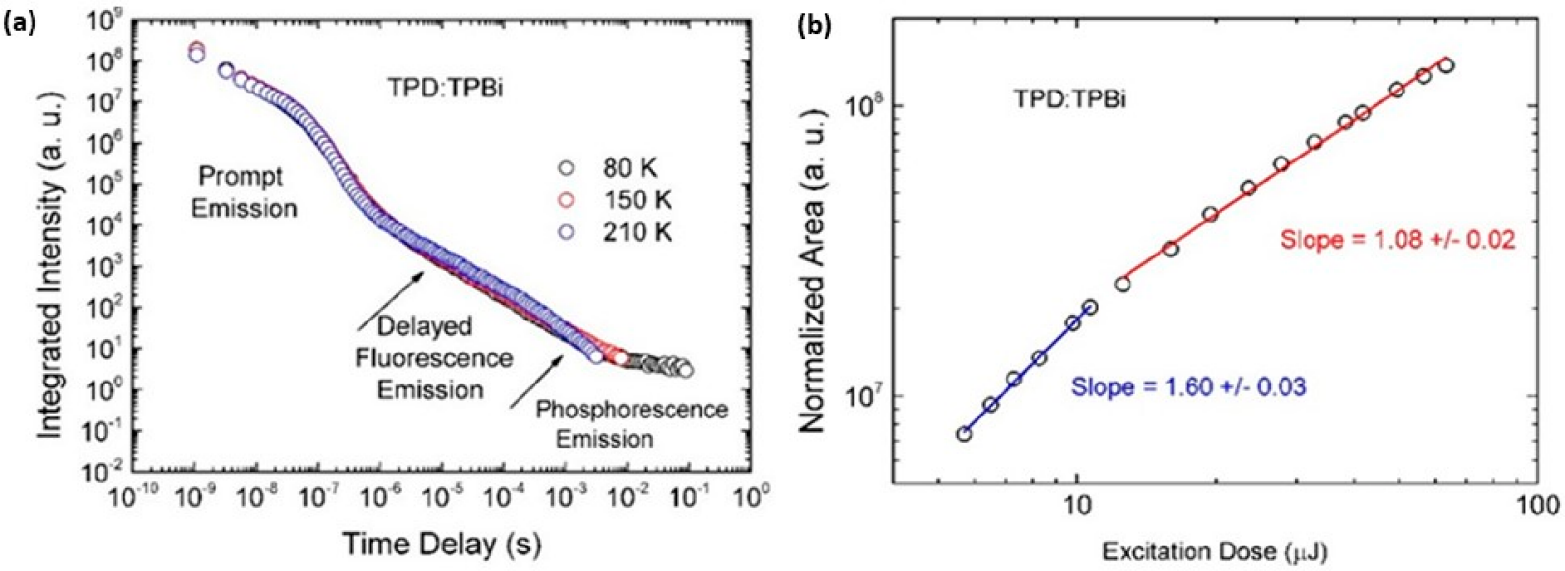
Photophysical analyses of TTA mechanism. (a) Time-resolved fluorescence decay curves at different temperatures for a TPD:TPBi exciplex blend, *N*,*N*′-bis(3-methylphenyl)-*N*,*N*′-diphenylbenzidine (TPD), 2,2′,2″-(1,3,5-benzinetriyl)-tris(1-phenyl-1-*H*-benzimidazole) (TPBi). (b) Integrated area as a function of the laser excitation dose (337 nm), collected in the delayed fluorescence region (TD = 2 μs and Ti = 20 μs). The intensity dependence shows a slope of 1.60 ± 0.03 at low excitation dose (<11 μJ), which turns to slope of 1.08 ± 0.02 at high excitation doses. This behavior strongly indicates a dominant TTA mechanism. Reprinted with permission from ref (125). Copyright © 2016, American Chemical Society.

It is worth mentioning that quadratic dependence of *I*_DF_ on excitation light intensity is mostly valid under low excitation densities. As excitation density increases, the triplet population [T] significantly rises, favoring the bimolecular process, TTA over the monomolecular quenching process.^244^ In the photophysical characterization of TTA systems, a method to ascertain if TTA is the underlying mechanism for DF involves examining the DF intensity in relation to the excitation dose. Generally, TTA complexes exhibit a slope close to 2 at low excitation doses, transitioning to a slope close to 1 at high excitation doses. In contrast, DF from TADF complexes typically shows a slope close to 1 at both low and high excitation doses. We demonstrated a detailed photophysical analysis,^125^ wherein DF from exciplex blends (donor and acceptor blends) was explored and the interplay between TTA and TADF as well as the excited states involved in those mechanisms was showcased (Figure 10).

**Figure 11:**
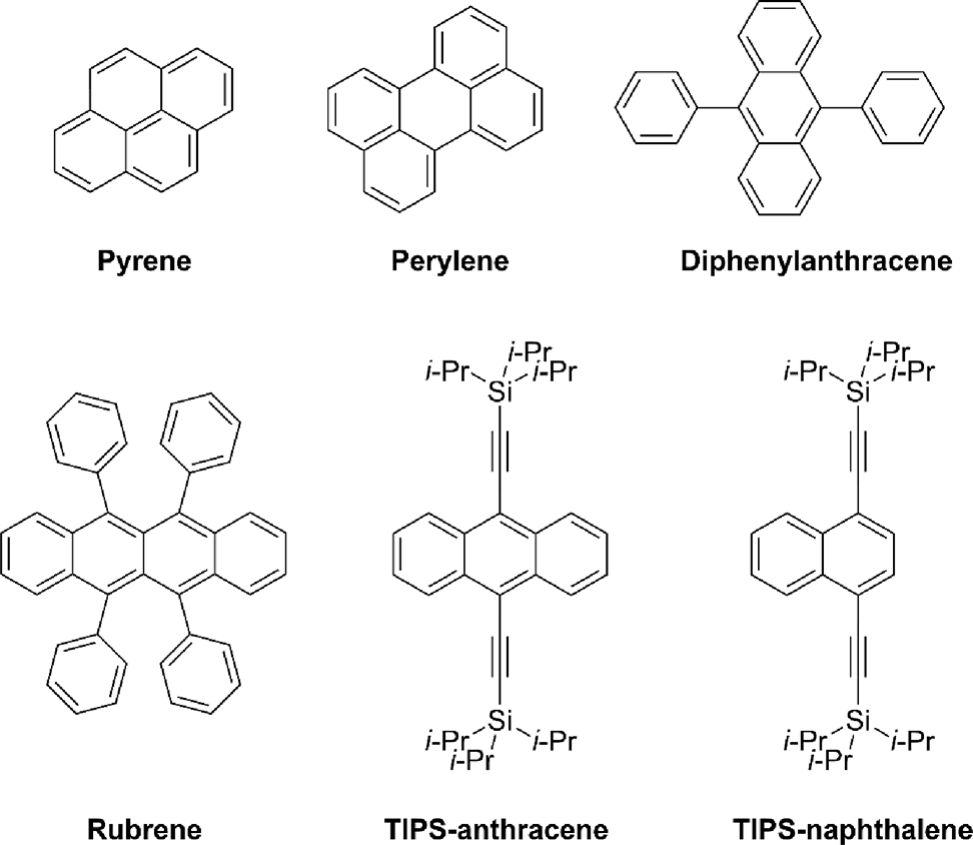
Molecular structure of mostly common molecules presenting TTA-UC: pyrene, perylene, diphenylanthracene (DPA), rubrene, 9,10-bis[((triisopropyl)silyl)ethynyl]anthracene (TIPS-anthracene), and 1,4-bis((triisopropylsilyl)ethynyl)naphthalene (TIPS-naphthalene).

The TTA mechanism has shown potential applications in OLEDs, photovoltaic technologies, and biomedical sciences.^245,246^ This great interest has led to the identification of a broad range of molecules undergoing this process. Figure 11 displays some examples of molecules that have been showing the mechanism, including molecules such as pyrene, perylene, rubrene, TIPS-anthracene, and TIPS-naphthalene.^247−249^

**Figure 12:**
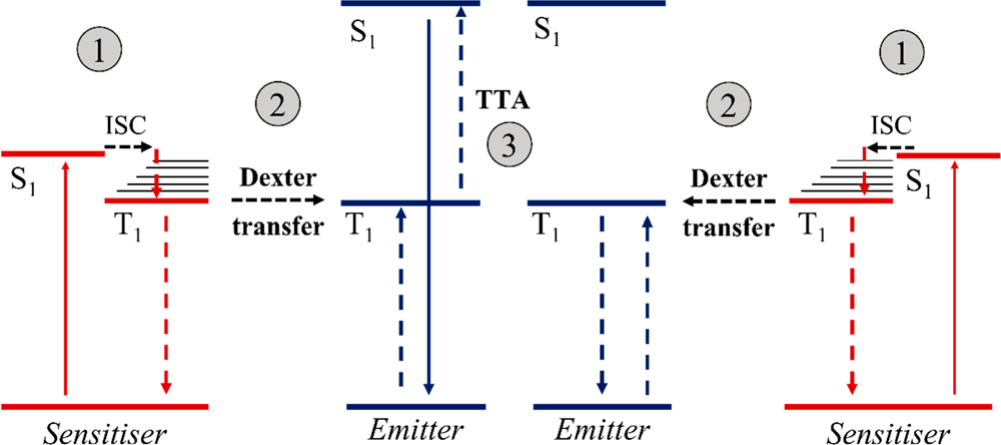
A simplified energy-level diagram illustrates the conventional TTA upconversion mechanism. In this process, two low-energy photons are absorbed by the sensitizers (1), transferred to the emitters through Dexter energy transfer (2), and ultimately transformed into a single high-energy photon through TTA (3).

To enhance the efficiency of the TTA mechanism, it is common to combine donor and acceptor species, also known as sensitizer and emitter, respectively. In this case, the mechanism can be described according to the simple scheme in Figure 12. The sensitizer is photoexcited, generating triplet states that are transferred to emitter molecules via Dexter energy transfer. Subsequent annihilation of two triplets results in an emitter singlet state, which then decays radiatively.^246,250,251^ Typical sensitizer choices include phosphorescent organometallic compounds, such as platinum or palladium porphyrins or organometallic phthalocyanines, as discussed in the phosphorescence section. Efficient TTA upconversion has been achieved in a solution, which has limited practical use.^252^ Solid-state TTA upconversion systems present a potential for real-world applications as they form films and layers; however, challenges persist in finding suitable host molecules for this system, as highlighted by *Evans et al*.^253^

### Applications

#### OLEDs

In the previous sections, we discussed TTA in OLEDs as a degradation mechanism found in phosphorescent and TADF-based devices. Due to the relatively long-lived triplet excitons within the phosphorescent and TADF emitters, TTA and triplet-polaron annihilation (TPA) easily occur between triplet excitons and between triplet exciton and polaron in devices. In this scenario, the excited states formed are highly energetic and can lead to molecular chemical decomposition. The formation of these high-energy excited states can be suppressed by using TTA emitters, i.e., emitters which show DF. Thus, the excited states formed in TTA emitters results in light emission, thereby reducing the risk of chemical decomposition in devices and potentially improving stability during the device operation. Therefore, many TTA materials have been explored as emitters in OLEDs.

**Figure 13:**
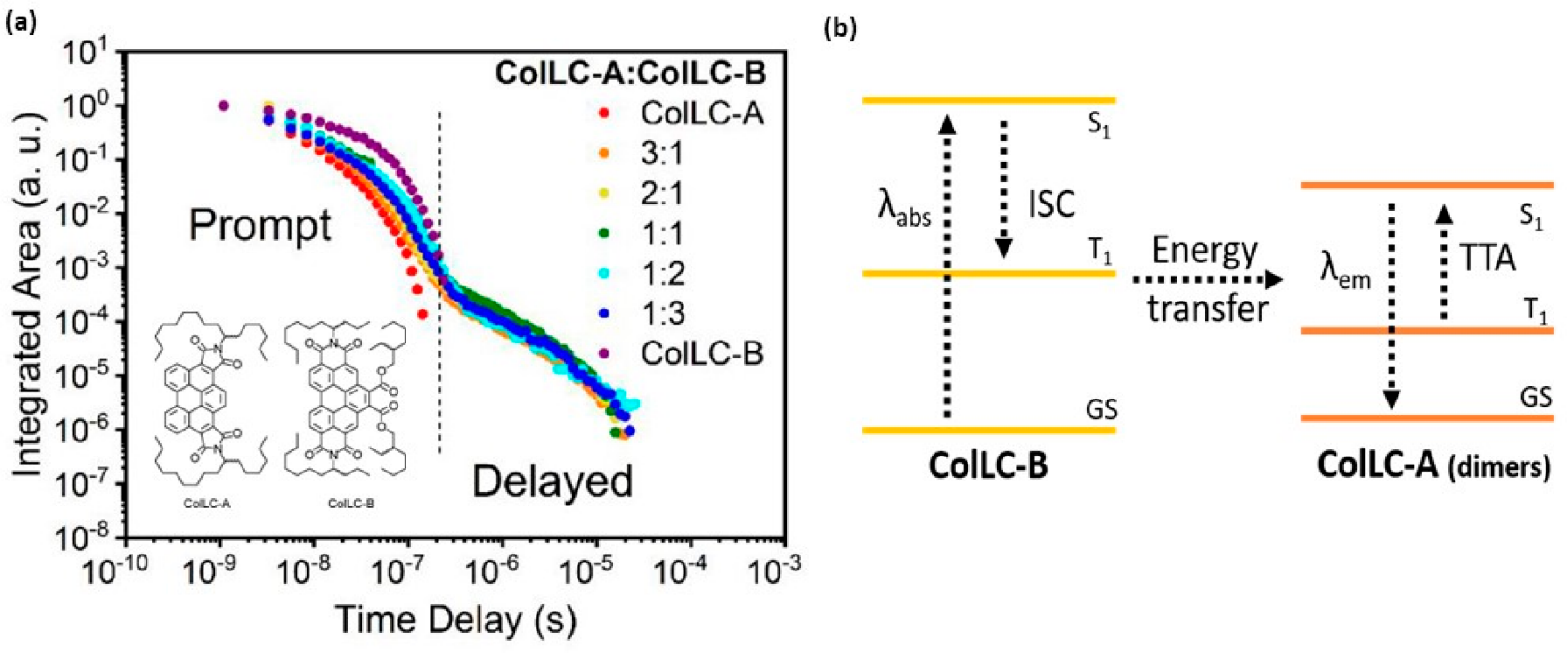
First observation of TTA in columnar liquid crystal films. (a) Time-resolved photoluminescence decay curves for ColLC-A, ColLC-B, and blend films at room temperature. Blends show DF via TTA mechanism, and the proposed mechanism is shown in (b). Reprinted with permission from ref (254). Copyright © 2022 American Chemical Society.

When TTA emitters are designed for OLEDs, the S_1_ state should be lower than the energy of a triplet pair and the energy of a triplet pair should be lower than the energy of the T_n_ state to avoid the quenching of the intermediate states by T_n_. This optimized energy alignment can be used in the emissive layer of OLEDs with a maximum theoretical IQE of 62.5%.^245^ TTA materials predominantly utilize anthracene as a foundational component, and additionally, materials derived from cyano-anthracene, nitrogen heterocycle-anthracene, imidazole, and phosphine oxide-anthracene have been explored as TTA emitters and reviewed by *Wong et al*.^245^ Moreover, different TTA systems have been explored; for example, we showed the first observation of TTA from a columnar liquid crystalline state^254^ (Figure 13). This work showed how a careful mix of liquid crystal materials with complementary functions can activate the TTA mechanism. Importantly, the observation of delayed fluorescence in the condensed viscous fluid state of liquid crystal materials, where molecules can be uniformly oriented by annealing, opens the possibility to use such materials as emissive layers of OLEDs to enhance light outcoupling as well as charge and exciton transport, achieving energy-efficient devices.

Regarding device performance, the TTA mechanism promises to advance the blue OLED problem, and blue TTA OLEDs with high EQE, efficient roll-off, and enhanced stability were developed.^255−258^ We highlight work by *Wang et al*.^259^ which developed two benzonitrile-anthracene derivatives named 3CzAnBzt and pCzAnBzt. The nondoped blue devices showed EQE above 10%, and the efficiency roll-off for 3CzAnBzt and pCzAnBzt was exceptionally low, with EQEs still remained 8% and 7% at the luminescence of 1000 cd m^–2^.

Elaboration of the appropriate host materials is also important for the fabrication of highly efficient OLEDs. Recently, *Fukagawa et al*.^260^ reported two anthracene-based TTA hosts (Spiro-FA and Spiro-FPA) by incorporating a spirobifluorene unit in the bulky molecular structures. Their strategy prevented the overlap of anthracene groups between adjacent molecules in the emissive layer promoting the upconversion of triplet into singlets. By using the novel hosts and 4,40-bis[4-(diphenylamino)styryl]biphenyl (BDAVBi) as the emitter for blue OLEDs, devices exhibiting EQEs of 5% and 7% and CIE coordinates of (0.17, 0.29) and (0.16, 0.23) were demonstrated. Moreover, molecular design routes on how to achieve both efficient blue emitters and ambipolar high triplet energy hosts by simple variation of molecular structure in derivatives of carbazole and nitrile-substituted 1,3,5-triphenylbenzene (TPB) were demonstrated.^261^ The introduction of the accepting nitrile groups in the para-position induced intensive DF via a TTA mechanism, while the meta-linkage led to ambipolar charge transport and higher triplet energies (2.82 eV), ideal for hosts. By utilization of the para-substituted derivative as an emitter and the meta-substituted isomer as the host, a deep-blue OLED with EQE of 14.1% was demonstrated.

#### Biomedical Uses and Sensing

**Figure 14:**
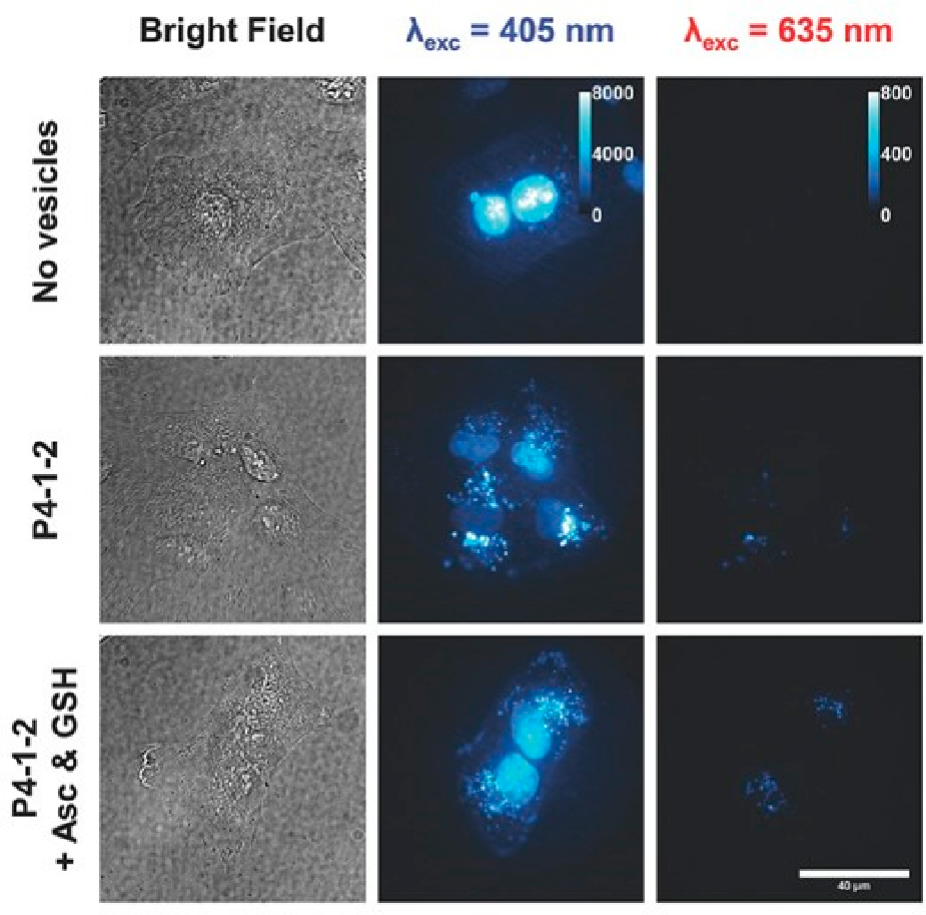
In vitro imaging using different TTA polymersomes. Imaging of upconverting polymersomes in living lung carcinoma cells in bright field mode (left column), with λ_exc_ = 405 nm (middle column) and with λ_exc_ = 635 nm (right column) with 100× magnification. Reprinted with permission from ref (270). © Copyright 2016 WILEY-VCH Verlag GmbH & Co. KGaA, Weinheim.

Similar to what is described in the previous sections, the TTA mechanism has also found much use in biomedical and sensing applications. As highlighted by *Wu et al*.,^262^ the exposure to high-intensity light has the potential to cause harm to cells, tissues, and other biomaterials. Consequently, the TTA mechanism offers a solution to these issues by enabling lower excitation intensities. Furthermore, TTA upconversion has proven to be versatile in terms of molecular tunability. When considering application, factors such as solubility, susceptibility to oxygen quenching, and the potential for metal toxicity at elevated dosages play an important role.^263^ In 2016, *Beverina and colleagues* presented a straightforward and versatile method for creating water-dispersible, self-assembled nanomicelles loaded with a pair of sensitizer/emitter chromophores. These nanomicelles exhibit efficient sensitized upconversion emission at low excitation power. In vitro fluorescence imaging experiments validated their high biocompatibility. Importantly, in this work, the potential cessation of the upconversion signal by an external stimulus shows promise in providing a direct and precise indication of the timing and location of the contents release within the biological specimen.^264^ The extent of the research has recently resulted in *Evans et al*.^265^ reporting the successful synthesis of water-dispersible nanoparticles with a diameter of 6 nm through emulsion polymerization. These nanoparticles exhibited TTA-UC activity in aerated aqueous media at room temperature. The application of TTA-UC nanoparticles has found relevance as one of the first examples of lifetime imaging in living cells, specifically using Chinese hamster ovary (CHO) cells under ambient physiological conditions. Numerous studies demonstrate the potential of TTA-UC in bioimaging.^264,266−269,271^Figure 14 illustrates an example of in vitro upconversion imaging of polymersomes in cancer cells.^270^

#### Other Applications

**Figure 15:**
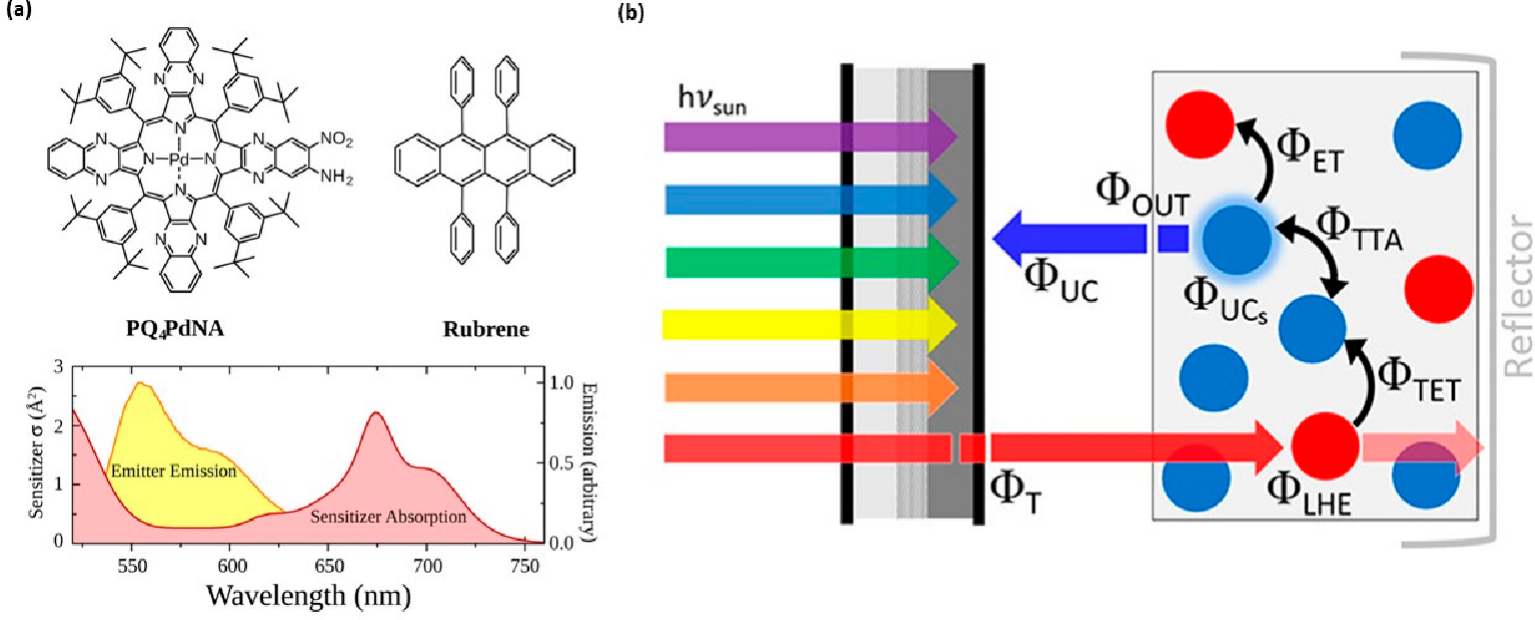
Use of TTA systems in solar cells. A combination of sensitizers with highly efficient TTA emitter can be used to build UC units to increase light-harvesting efficiency in solar cells. (a) A typical sensitizer (PQ4PdNA) and emitter (rubrene) species used in these UC units. Graph shows absorption spectrum of PQ4PdNA compared with the emission spectrum of rubrene. The window in the absorption spectrum allows a large proportion of rubrene fluorescence to escape the upconversion medium. Reprinted with permission from ref (279). Copyright © 2017, American Chemical Society. (b) Diagram depicting the events and quantum yields for TTA UC in an optically coupled UC solar cell with the sensitizer and annihilator molecules depicted in red and blue, respectively. Reprinted with permission from ref (20). Copyright © 2021, American Chemical Society.

Due to the tunable excitation and emission wavelength properties of TTA, along with its strong absorption of visible light and high upconversion quantum yields, TTA systems are particularly pertinent in energy applications such as solar cells and photocatalysis. Upconversion of low-energy photons into high-energy light can increase the efficiency of solar devices by converting photons with energies below the energy absorption threshold (which initially would be wasted) into radiation that can be utilized by the solar cell. We highlight the pioneering work by *Schmidt et al*.,^272^ which reported the first integrated hydrogenated amorphous silicon (a-Si:H) and TTA photovoltaic device. They used porphyrins as sensitizers in combination with the highly efficient TTA emitter rubrene to build the UC unit placed behind the a-Si:H solar cell, resulting in increased light-harvesting efficiency (Figure 15). A similar principle has been used in different solar cells systems^233,273−275^ including the emerging perovskite solar cells, which have their absorption mostly limited to the visible range. In 2020, *Kimizuka et al*.^276^ showed the first example of endowing perovskite solar cells with NIR sensitivity by using solid films showing NIR-to-vis UC based on TTA. A notable TTA efficiency (4% at an excitation intensity of 125 W/cm^2^) was achieved by sensitizing a rubrene (acceptor) triplet with an osmium complex donor having singlet-to-triplet absorption in the NIR range. Incorporating the TTA-UC film behind a semitransparent perovskite solar cell results in generation of photocurrent under excitation at 938 nm. To explore additional research on the application of TTA systems in solar cells we guide the readers to the reviews.^250,277,278^

In the photocatalysis field, we highlight an environmental application pioneered by *Kim et al*.^280^ Their study showed photocatalytic decomposition of an indoor air pollutant, acetaldehyde, using low-energy, sub-bandgap photons harnessed through sensitized TTA. Most indoor light irradiation is not sufficient for photocatalysis, and to solve this problem they designed a sub-bandgap photocatalyst device with a TTA rubbery polymer film to upconvert sub-bandgap photons combined with a nanodiamond (ND)-loaded WO_3_ as a visible-light photocatalyst composite. They showed that effective decomposition of acetaldehyde was achieved using ND/WO_3_ (*E*_g_ = 2.8 eV) coupled with TTA polymer films that emit blue photons (λ_Em_ = 425 nm, 2.92 eV) upconverted from green photons (λ_Ex_ = 532 nm, 2.33 eV), which are usually wasted in most environmental photocatalysis. This promising indoor air-purification approach has been lately explored in more complex dual layers of UC systems composed of multiple sensitizers.^281^

#### Limitations & Perspectives

**Figure 16:**
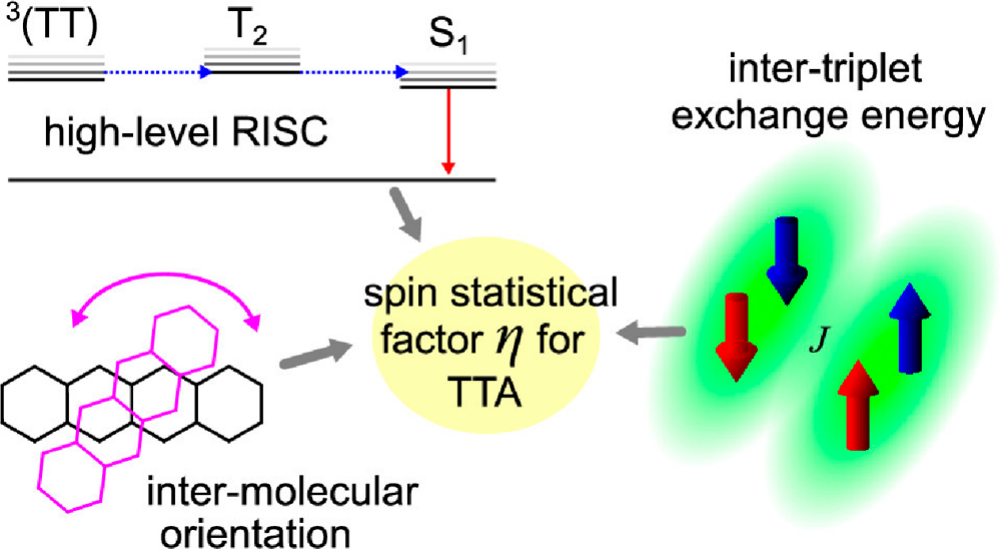
Illustrative diagram displaying the variables impacting the spin statistical factor, η, that gives the probability that a bright singlet state is formed from a pair of annihilating triplet states. Using solid rubrene as a model system *Clark et al*. provided an updated model framework with which to understand the spin statistics of TTA upconversion. Reprinted with permission from ref (282). Copyright © 2021, American Chemical Society.

The first limitation we would like to highlight revolves around the comprehension of the TTA mechanism. The probability that a pair of annihilating triplet excitons results in a singlet exciton is given by the spin statistical factor, η, with 0 ≤ η ≤ 1. However, despite its fundamental importance, the triplet–triplet interactions that govern the value of η are not, in general, fully understood or appreciated.^23^ As a result, several potential strategies for designing materials with a high value of η have been largely overlooked to date. Recently, *Bossanyi et al*.^282^ investigated the triplet-pair character, energy levels, internal conversion rate constants, and reverse intersystem crossing in rubrene, the most common acceptor molecule for near-infrared-to-visible TTA upconversion (Figure 16). Based on experimental results, they presented an updated model for the spin statistics of upconversion that includes the effects of intertriplet exchange coupling and orientation as well as internal conversion rate constants, energy levels, and reverse intersystem crossing. They found that variations in exchange energy and orientation can tune the spin statistical factor η within the range 2/5 ≤ η ≤ 2/3, but that careful optimization of the S_1_, T_2_, and T_1_ energy levels may allow η to reach unity, thereby by passing such considerations. Thus, this work points the way toward strategies for exceeding the spin statistical limit of TTA.

Regarding OLEDs, one limitation is the fabrication method. TTA-based OLEDs are almost all fabricated by a vacuum-evaporation approach, and solution-processed TTA devices still remain a great challenge due to the rigidity and large π-conjugation of this type of materials. Exploring this limitation, *Liu et al*.^283^ designed a novel blue TTA emitter with a fan-shape torsional molecular structure, namely, TbuPyB, which is based on benzene as core and three *tert*-butylpyrenes as arm. The new emitter shows PLQY of 74% in neat film, and the solution-processed nondoped devices exhibited a blue emission peak at 470 nm with an impressive EQE of 10.65%. This molecular strategy certainly opens up opportunities for the development of TTA-based OLEDs using a wet fabrication process.

## Conclusion

Phosphorescence, Thermally Activated Delayed Fluorescence, and Triplet–Triplet Annihilation have been growing in prevalence within the scientific literature, and their range of applications extended in the past decades. Here, we explored their working principles and discussed recent pivotal advancements concerning their underlying mechanisms, molecular design, applications in OLED technology, and biomedical and sensing applications as well as other emerging applications. We emphasized that although each mechanism has distinct limitations, there are overarching challenges shared among all three, as all the mechanisms involve triplet states. We advocate for prioritizing studies aimed at overcoming these central challenges to pave the way for fully harnessing the potential of these materials.
